# Infectious disease research in forcibly displaced populations: A systematic review in low- and middle-income host countries

**DOI:** 10.1016/j.jmh.2025.100341

**Published:** 2025-06-27

**Authors:** Neila Gross, Maia C. Tarnas, Rashmina J. Sayeeda, Carly Ching, David Flynn, Muhammad H Zaman

**Affiliations:** a Department of Materials Science and Engineering, Boston University, Boston, MA, USA; b Department of Population Health and Disease Prevention, University of California Irvine, Irvine, CA, USA; c Department of Neuroscience, Boston University, Boston, MA, USA; d Department of Biomedical Engineering, Boston University, Boston, MA, USA; e Department of Medical Sciences and Education, Boston University, Boston, MA, USA; f Center on Forced Displacement, Boston University, Boston, MA, USA

**Keywords:** Infectious disease, Forcibly displaced people, Research practices, Ethics

## Abstract

**Background:**

Infectious disease research is essential for disease prevention and management within refugee camps and informal settlements. The objective of this study is to identify the characteristics of existing infectious disease research in these settings and to assess stated research challenges, ethical considerations, and studied interventions within these studies.

**Methods:**

This is a systematic review of forty primary studies focused on infectious disease research conducted among displaced populations. Included studies are published in English between 1995 and 2023. Three databases were searched, PubMed, Embase, and Web of Science, and this review was registered with PROSPERO (CRD42023461567). The risk of bias of the studies was assessed using the Mixed Methods Appraisal Tool.

**Results:**

85 % of studies (*n* = 34) researched an intervention for infectious disease prevention or control and 70 % of studies (*n* = 28) were randomized controlled trials. 75 % of studies were located in Bangladesh (*n* = 15) or Pakistan (*n* = 15). 40 % of studies focused on diarrheal diseases (*n* = 16) and 28 % on malaria (*n* = 11). Common identified research challenges included population mobility, limited external validity, and low recruitment. No studies included the community in the initial study conception or investigated the research impact on the community. Community involvement was often through community health workers (45 %). Of the 18 studies that studied a resource-based intervention, 20 % explicitly noted that the intervention was unsustainable.

**Discussion:**

While guidelines for conducting research in displaced settings exist, there are gaps in their utilization. We identified a disconnect between where displaced individuals reside and where research is conducted, as well as a prioritization of particular infectious diseases. Researchers identified numerous challenges in conducting research in these settings, though the community was rarely involved in the research. Context-specific considerations and community involvement are vital in research with displaced communities.

**Funding:**

Wellcome Trust (Contract Number C-010,656).

## 1 Introduction

The United Nations Refugee Agency (UNHCR) estimates that in 2022, 108.4 million people remained forcibly displaced. This included both refugees (people who are housed outside their country of citizenship) and internally displaced persons (communities that are displaced within their own countries). Of the 29.4 million documented as refugees, 76 % are hosted in low- and middle-income countries (LMICs) (UNHCR 2023). The number of displaced individuals is expected to grow due to increasing poverty, insecurity, and declining access to essential services due to conflict and climate change-related environmental disasters (WHO 2022). Displaced individuals are more susceptible to infectious diseases, often stemming from poor housing, insufficient access to healthcare and water, sanitation, and hygiene (WASH) infrastructure, environmental exposures, and overcrowding (Taha et al., 2023). Given this, research on infectious disease prevention, management, and control within the context of refugee camps and informal settlements is essential for enhancing public health preparedness and response. However, it is critical that such research does not further increase the harm or vulnerability of groups that already face compounding marginalization (Ching and Zaman, 2023).

Several organizations, including the World Health Organization (WHO) and the Refugee Studies Center, have outlined frameworks for conducting ethical research with refugees or displaced persons (MacFarlane et al., 2023; Refugee Studies Centre 2007). Other humanitarian organizations that operate frequently in refugee camps, such as United Nations Children’s Fund (UNICEF) and Médecins Sans Frontières, have broad ethical research guidelines that are not specific to working with displaced communities (UNICEF 2021; MSF Ethics Review Board 2013). Most of these guidelines and frameworks emphasize participatory research methods, or the involvement of displaced people throughout the research process. The benefits of community-based participatory research and other similar participatory methods are vast: by establishing an equitable partnership between the researchers and community, the research can address specific issues identified by the community in an appropriate, culturally competent, and sustainable fashion. Central to this process is the involvement of the community throughout the entire research process, which strengthens the research quality within the specific context and ensures that the research outcome is relevant and desired by the community (WHO 2022). Though there is apparent agreement, at least in academic and humanitarian circles, in the overarching framing of how research involving refugees and other types of displaced people *should* be conducted, the extent to which this is actually done is unclear (Rustage et al., 2021; Müller-Funk, 2021).

These ethical research considerations are especially critical in LMICs, where the health sciences have a notorious history of extractive research (Abimbola, 2018; Edejer, 1999; Chu et al., 2014). Historically (and to some degree, currently), there has been a pattern of funding awards granted to researchers from high income countries to conduct health research in LMICs. These research teams often lack the proper training to soundly navigate diverse ethical considerations to ensure the safety and integrity of their study population, when this is even considered. In many of these cases, the research operation concludes when it has maximized the benefit to the researchers, not the community (Agarwal et al., 2024). In host countries that neighbor refugees’ country of origin, there may be increased scrutiny of refugees because of ongoing conflict, sociopolitical tensions, and xenophobia; infectious disease research must be aware of its potential to widen such divides (Müller-Funk, 2021). Infectious disease research in displaced settings often involves heightened urgency, public health surveillance, and potential stigmatization of individuals or groups—factors that can further marginalize already vulnerable populations (WHO 2022; Taha et al., 2023). These dynamics call for specialized ethical considerations, including culturally responsive consent processes, the protection of community reputation, data security, and meaningful involvement of displaced people in research design and dissemination (Rustage et al., 2021; Müller-Funk, 2021). While notable ethical frameworks for such work have been published, it is unclear the degree to which they have been followed in these settings.

In this systematic review, we aim to identify current research on the prevention, treatment, and management of infectious diseases within refugee camps and informal settlements in the ten LMICs that host refugees and other types of forcibly displaced people (UNHCR 2023; UNHCR 2023). We will assess the stated associated challenges in conducting this research, ethical considerations throughout the research process (including community involvement), and studied interventions (including their feasibility) within these studies. In doing so, we seek to provide a comprehensive overview of *what* research has been done in these settings, *how* it has been done, and *where* specific challenges and gaps arise.

## 2 Methods

We conducted a systematic review of infectious disease prevention, treatment, and control research in refugee camps and informal settlements in the top ten refugee-hosting LMICs in 2023, as defined by the UNHCR (UNHCR 2023; UNHCR 2023). These countries are: 1. Iran (3.4 million refugees); 2. Pakistan (2.1 million); 3. Uganda (1.5 million); 4. Bangladesh (1 million); 5. Sudan (900,000); 6. Ethiopia (900,000); 7. Lebanon (800,000); 8. Democratic Republic of Congo (DRC) (500,000); 9. Kenya (500,000); and 10. Cameroon (500,000). This review follows PRISMA guidelines and is registered on PROSPERO (CRD42023461567).

### 2.1 Search strategy and inclusion criteria

We searched PubMed, Embase, and Web of Science using MeSH terms and select free-text keywords (complete search strategy available in the **appendix**) on August 4th, 2023. Such databases largely cover biomedical and life sciences literature, as well as social science and humanities literature. Search terms were refined iteratively to maximize relevant retrieval. A full protocol can be found on the PROSPERO website or in the appendix; this protocol and full search strategy were developed in collaboration with a Senior Research Librarian (DF). We did not use a date or language filter in our initial search. Title-abstract and full-text screenings were conducted blindly and independently by three authors (NG, MCT, and RJS) using the web-based application Rayyan. Any discrepancies were discussed until consensus was reached. To be included, studies had to be primary research of any study design, explicitly include refugees or be conducted in a refugee camp, informal settlement, or displaced people-serving hospital, and focus directly on at least one infectious disease. We included informal settlements because an estimated 88 % of refugees live outside of camps, often in substandard urban dwellings (UNHCR 2021). We did not impose any limitation on infectious disease type or age of affected population. Papers that focused on topics that may have a downstream, indirect, or implied effect on infectious diseases were excluded (Ouzzani et al., 2016).

### 2.2 Data extraction and analysis

Following the full-text review, we extracted data on study location, disease focus, participants, methodology, ethical considerations, stated challenges, intervention (if appropriate), and any other relevant information into a standardized spreadsheet. Research or study challenges were extracted if researchers specifically noted them as a challenge or limitation. For ethical considerations, we extracted information regarding community involvement, consent, ethical approvals (including approval from host country or other local authorities), context-specific adaptations to research materials, length of involvement with community, and funding source. We were particularly interested in how researchers involved the study population during the study conception and design. To assess this, we pulled information on community member involvement throughout the research process, including development, design, and execution. Involvement in research development and design was defined as explicit inclusion of the community in developing the research question and methodology, or iteratively designing the research with the guidance of community members. Community members were considered to be involved in the research execution if they worked in data collection (e.g., as a community health worker, translator, field worker, etc.) or in data analysis. Lastly, to measure intervention sustainability, we modeled our assessment on Proctor’s Implementation Outcomes Taxonomy, which outlines eight key outcomes to evaluate the success of health interventions: acceptability, adoption, appropriateness, feasibility, fidelity, implementation cost, penetration, and sustainability (Proctor et al., 2010). Initial acceptability, appropriateness, and adoption were assessed by identifying whether studies involved the community in research development and design, thereby tailoring the intervention to local needs. We likewise considered that the inclusion of community members in performing the research - and therefore the intervention - reflects a step toward embedding the intervention in local structures, suggesting operational feasibility within the specific context and enhanced fidelity, as their delivery may be more culturally appropriate and contextually consistent. Finally, we assessed sustainability based on whether the interventions were likely to be maintained and continue to produce benefits beyond the study period. Specifically, we examined reported indicators of sustainability (e.g., ongoing delivery plans), implementation cost, appropriateness (fit for the context), and feasibility (practicality of implementation). Ultimately this is determined by whether: (1) the intervention was designed or adapted with long-term use and context in mind, (2) there were explicit plans, structures, or resources (e.g., funding, training, policy integration) to support its ongoing delivery, and in particular, (3) the intervention was still in place or operationally viable beyond the study period. To avoid reporting bias, two authors extracted information independently (NG and MCT) and compared for discrepancies.

Following data extraction, study characteristics (e.g., location, date, diseases of focus, etc.) were compiled via basic descriptive statistics. We then synthesized and thematically grouped data by identified challenges, ethical considerations and community involvement, and intervention feasibility and sustainability as stated above. We only included challenges explicitly reported by study authors and use these to iteratively develop a list of common challenges. When analyzing data on community involvement, we combined deductive and inductive approaches to assess involvement across pre-identified areas of the research process (e.g., methodology) while also ensuring that we captured all stated involvement by iteratively expanding this definition based on the data. Lastly, intervention data was first synthesized based on intervention type and purpose; we also assessed how the intervention was provided, including long-term provision, and associated costs. As with the community involvement, we combined deductive and inductive approaches to analyzing information on intervention sustainability as described above, and iteratively grouped findings thematically, including cost-effectiveness, availability of material, and cultural considerations.

### 2.3 Bias assessments

We assessed studies’ risk of bias using the Mixed Methods Appraisal Tool (MMAT) (Hong et al., 2018). For each study design type, the MMAT poses five criteria to assess bias risk. These five criteria differed by study design and can be seen in Figure S1. Studies that met at least four out of five criteria were considered high quality (i.e., low risk of bias, see supplementary information) (Chu et al., 2020). Two authors (MCT and NG) independently completed this analysis, and discrepancies were resolved by comparing textual evidence.

We also assessed the studies for publication bias. Outcomes were defined as positive, negative, and mixed (including both positive and negative results). Medical interventions were considered positive if there was a statistical difference in favor of the intervention and placebo groups. Survey studies had a positive outcome if researchers were able to properly administer and complete the survey and found significant correlations resulting from the survey.

### 2.4 Role of the funding source

The funder of the study had no role in study design, data collection, data analysis, or data interpretation.

## 3 Results

### 3.1 Screening results

**Fig. 1:**
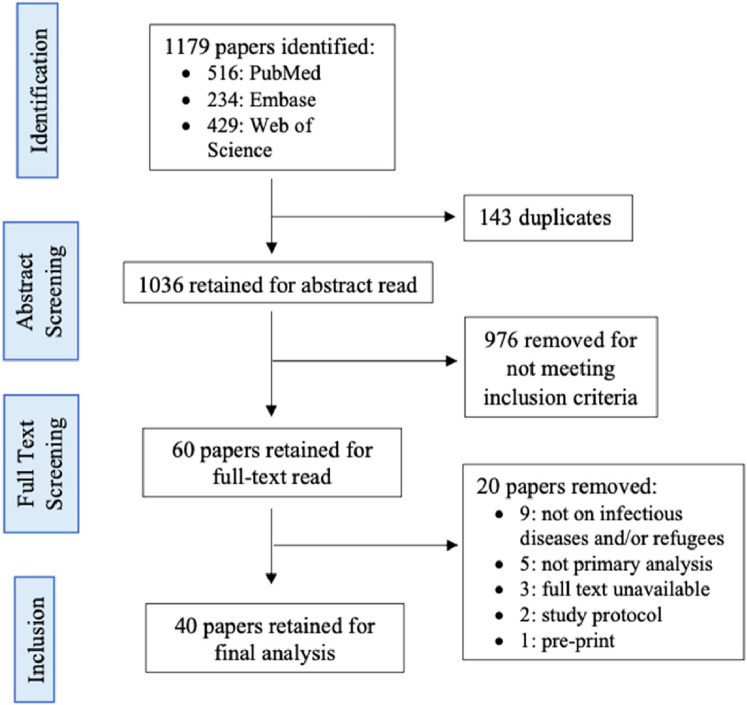
Study selection flowchart.

Our initial searches returned 1179 records (Fig. 1). Of the 1036 that remained following deduplication, 976 were removed following title and abstract review. Twenty papers were excluded after full-text screening. Based on this, 40 papers met the review’s inclusion criteria (Fig. 1).

### 3.2 Study characteristics

The included studies were published between 1995 and 2023, and most were located in Bangladesh (*n* = 15) (Rego et al., 2021; Zohura et al., 2022; Ali et al., 2021; ATM Mohiuddin Chowdhury et al., 2022; Brooks et al., 2005; ATM Mohiuddin Chowdhury et al., 2022; Faruque et al., 2021; Larson et al., 2010; Luoto et al., 2011; Qadri et al., 2003; Rahman et al., 2001; Mitra et al., 1997; Najnin et al., 2019; Pickering et al., 2015; Pickering et al., 2019) and Pakistan (*n* = 15) (Brown et al., 2020; Rowland et al., 2001; Rowland et al., 1999; Graham et al., 2002; Howard et al., 2011; Kolaczinski et al., 2004; Kolaczinski et al., 2012; Leslie et al., 2004; Luby et al., 2005; Luby et al., 2004; Murphy et al., 1996; Rowland et al., 1996; Rowland et al., 2004; Rowland and Durrani, 1999; Owais et al., 2011) (Table 1). Table 1 demonstrates the differences in research location and disease studied. No studies were conducted in Lebanon, Iran, or Cameroon. Sixteen studies (Mekonnen et al., 2023; Rego et al., 2021; Zohura et al., 2022; Ali et al., 2021; Brooks et al., 2005; Faruque et al., 2021; Larson et al., 2010; Luby et al., 2005; Luby et al., 2004; Luoto et al., 2011; Murphy et al., 1996; Qadri et al., 2003; Rahman et al., 2001; Mitra et al., 1997; Pickering et al., 2015; Pickering et al., 2019) (40 %) focused on diarrheal diseases and 11^31,35–38,41,42,44,52–54^ (28 %) on malaria.

**Table 1:** Table of study location, year, and disease of focus.

	Diarrhea	Malaria	HIV/STD	COVID-19	Acute resp. infection	Pneumonia	Other	Total
Bangladesh	**12**	**0**	**0**	**2**	**3**	**1**	**0**	**18**
1995–2000	1	–	–	–	1	1	–	**2**
2001–2006	3	–	–	–	1	–	–	**5**
2007–2012	2	–	–	–	–	–	–	**2**
2013–2018	1	–	–	–	–	–	–	**1**
2019–2023	5	–	–	2	1	–	–	**9**
Pakistan	**3**	**10**	**0**	**0**	**1**	**1**	**3**	**18**
1995–2000	1	3	–	–	–	–	–	**4**
2001–2006	2	5	–	–	1	–	2*	**10**
2007–2012	–	2	–	–	–	–	1⁎⁎	**3**
2013–2018	–	–	–	–	–	–	–	**0**
2019–2023	–	–	–	–	–	1	–	**1**
Uganda	**0**	**0**	**1**	**2**	**0**	**0**	**0**	**3**
1995–2000	–	–	–	–	–	–	–	**0**
2001–2006	–	–	–	–	–	–	–	**0**
2007–2012	–	–	–	–	–	–	–	**0**
2013–2018	–	–	1	–	–	–	–	**1**
2019–2023	–	–	–	2	–	–	–	**2**
Kenya	**0**	**0**	**4**	**0**	**0**	**0**	**0**	**4**
1995–2000	–	–	–	–	–	–	–	**0**
2001–2006	–	–	2	–	–	–	–	**2**
2007–2012	–	–	–	–	–	–	–	**0**
2013–2018	–	–	1	–	–	–	–	**1**
2019–2023	–	–	1	–	–	–	–	**1**
DRC	**0**	**1**	**0**	**0**	**0**	**0**	**0**	**1**
1995–2000	–	1	–	–	–	–	–	**1**
2001–2006	–	–	–	–	–	–	–	**0**
2007–2012	–	–	–	–	–	–	–	**0**
2013–2018	–	–	–	–	–	–	–	**0**
2019–2023	–	–	–	–	–	–	–	**0**
Sudan	**0**	**0**	**0**	**0**	**0**	**0**	**1**	**1**
1995–2000	–	–	–	–	–	–	–	**0**
2001–2006	–	–	–	–	–	–	–	**0**
2007–2012	–	–	–	–	–	–	–	**0**
2013–2018	–	–	–	–	–	–	–	**0**
2019–2023	–	–	–	–	–	–	1⁎⁎⁎	**1**
Ethiopia	**1**	**0**	**0**	**0**	**0**	**0**	**0**	**1**
1995–2000	–	–	–	–	–	–	–	**0**
2001–2006	–	–	–	–	–	–	–	**0**
2007–2012	–	–	–	–	–	–	–	**0**
2013–2018	–	–	–	–	–	–	–	**0**
2019–2023	1	–	–	–	–	–	–	**1**
Total	**17**	**13**	**5**	**4**	**4**	**2**	**4**	

⁎ : impetigo and leishmaniasis.

⁎⁎ : diphtheria, tetanus, pertussis, and hepatitis B.

⁎⁎⁎ : trachoma.

Most studies researched an intervention (85 %, *n* = 34), (Mekonnen et al., 2023; Zohura et al., 2022; Ali et al., 2021; ATM Mohiuddin Chowdhury et al., 2022; Brooks et al., 2005; ATM Mohiuddin Chowdhury et al., 2022; Wolday et al., 1995; van der Kop et al., 2018; Stein et al., 2022; Rowland et al., 2001; Rowland et al., 1999; Graham et al., 2002; Howard et al., 2011; Kaul et al., 2004; Kimani et al., 2006; Kolaczinski et al., 2004; Kolaczinski et al., 2012; Larson et al., 2010; Leslie et al., 2004; Luby et al., 2005; Luby et al., 2004; Luoto et al., 2011; Murphy et al., 1996; Ngure et al., 2022; Qadri et al., 2003; Rahman et al., 2001; Rowland et al., 1996; Rowland et al., 2004; Rowland and Durrani, 1999; Mitra et al., 1997; Najnin et al., 2019; Owais et al., 2011; Pickering et al., 2015; Pickering et al., 2019) and most (70 %, *n* = 28) (Mekonnen et al., 2023; Ali et al., 2021; ATM Mohiuddin Chowdhury et al., 2022; Brooks et al., 2005; ATM Mohiuddin Chowdhury et al., 2022; van der Kop et al., 2018; Rowland et al., 1999; Graham et al., 2002; Howard et al., 2011; Kaul et al., 2004; Kimani et al., 2006; Kolaczinski et al., 2012; Larson et al., 2010; Leslie et al., 2004; Luby et al., 2005; Luby et al., 2004; Luoto et al., 2011; Ngure et al., 2022; Qadri et al., 2003; Rahman et al., 2001; Rowland et al., 1996; Rowland et al., 2004; Rowland and Durrani, 1999; Mitra et al., 1997; Najnin et al., 2019; Owais et al., 2011; Pickering et al., 2015; Pickering et al., 2019) were randomized controlled trials (RCTs) (Table 2). Qualitative or mixed-methods work was rarely seen in the included studies.

**Table 2:** Details of studies included in this review.

Citation	Location	Disease focus	Study design	Topic of study/intervention
Larson et al. (Larson et al., 2010)	Bangladesh	Diarrhea	RCT	Zinc supplementation after diarrheal treatment
Rego et al. (Rego et al., 2021)	Bangladesh	Diarrhea	Cross-sectional	Comparing diarrhea measurement tools
Zohura et al. (Zohura et al., 2022)	Bangladesh	Diarrhea (cholera)	Mixed methods	Development of a cholera response program
Ali et al. (Ali et al., 2021)	Bangladesh	Diarrhea (cholera)	RCT	Cholera vaccine effectiveness
Mohiuddin Chowdhury et al. (ATM Mohiuddin Chowdhury et al., 2022)	Bangladesh	COVID-19	RCT	Clinical recovery from COVID-19
Brooks et al. (Brooks et al., 2005)	Bangladesh	Pneumonia and diarrhea	RCT	Zinc supplementation to reduce disease incidence
Mohiuddin Chowdhury et al. (ATM Mohiuddin Chowdhury et al., 2022)	Bangladesh	COVID-19	RCT	Treatment of severe COVID-19
Faruque et al. (Faruque et al., 2021)	Bangladesh	Diarrhea	Cross-sectional	Characteristic of patients hospitalized with diarrhea
Qadri et al. (Qadri et al., 2003)	Bangladesh	Diarrhea	RCT	Vaccine safety and immunogenicity
Rahman et al. (Rahman et al., 2001)	Bangladesh	Diarrhea and ARI	RCT	Zinc and vitamin A supplementation
Luoto et al. (Luoto et al., 2011)	Bangladesh	Diarrhea	RCT	Water treatment products and education
Pickering et al. (Pickering et al., 2015)	Bangladesh	Diarrhea	RCT	Water treatment methods
Pickering et al. (Pickering et al., 2019)	Bangladesh	Diarrhea	RCT	In-line drinking water chlorination
Mitra et al. (Mitra et al., 1997)	Bangladesh	Diarrhea, dysentery, ARI	RCT	Long-term oral iron supplementation
Najnin et al. (Najnin et al., 2019)	Bangladesh	ARI	RCT	Handwashing intervention
Owais et al. (Owais et al., 2011)	Pakistan	DTP/Hep B vaccine	RCT	Low-literacy educational intervention
Brown et al. (Brown et al., 2020)	Pakistan	Pneumonia	Case-control	Associations with environmental factors
Rowland et al. (Rowland et al., 1996)	Pakistan	Malaria	RCT	Pyrethroid-impregnated bed nets
Rowland et al. (Rowland et al., 2004)	Pakistan	Malaria	RCT	DEET mosquito repellent soap
Rowland and Durrani (Rowland and Durrani, 1999)	Pakistan	Malaria	RCT	Malaria treatment
Rowland et al. (Rowland et al., 2001)	Pakistan	Malaria	Randomized crossover trial	Insecticide treatment of cattle
Rowland et al. (Rowland et al., 1999)	Pakistan	Malaria	RCT	Insecticide-treated chaddars and top-sheets
Graham et al. (Graham et al., 2002)	Pakistan	Malaria	RCT	Comparison of insecticides for top-sheet treatment
Howard et al. (Howard et al., 2011)	Pakistan	Malaria	RCT	Malaria treatment
Kolaczinski et al. (Kolaczinski et al., 2004)	Pakistan	Malaria and CL	Case study	Shift to subsided sale of bed nets
Kolaczinski et al. (Kolaczinski et al., 2012)	Pakistan	Malaria	RCT	Malaria treatment
Leslie et al. (Leslie et al., 2004)	Pakistan	Malaria	RCT	Compliance with malaria treatment
Luby et al. (Luby et al., 2005)	Pakistan	Diarrhea, impetigo, ARI	RCT	Handwashing promotion with soap
Luby et al. (Luby et al., 2004)	Pakistan	Diarrhea	RCT	Handwashing promotion with soap
Murphy et al. (Murphy et al., 1996)	Pakistan	Diarrhea	Non-randomized controlled trial	Trial of wheat-based oral rehydration solution
Logie et al. (Logie et al., 2023)	Uganda	COVID-19	Cross-sectional	COVID-19 vaccination acceptability
Stein et al. (Stein et al., 2022)	Uganda	COVID-19	Mixed methods	Impact of one-time cash transfer
O’Laughlin et al. (O’Laughlin et al., 2016)	Uganda	HIV	Cross-sectional	Associations with HIV diagnosis
Kaul et al. (Kaul et al., 2004)	Kenya	HIV / STI	RCT	Antibiotic prophylaxis for STIs and HIV
Kimani et al. (Kimani et al., 2006)	Kenya	Malaria	RCT	Insecticide-treated clothes
Ngure et al. (Ngure et al., 2022)	Kenya	HIV	RCT	6-month PrEP dispensing with self-HIV testing
van der Kop et al. (van der Kop et al., 2018)	Kenya	HIV	RCT	Patient retention in HIV care
Wolday et al. (Wolday et al., 1995)	DRC	Malaria	Cross-sectional	Measuring the resistance of *P. falciparum* to antimalarials
Sanders et al. (Sanders et al., 2019)	Sudan	Trachoma	Cross-sectional	Measuring trachoma prevalence
Mekonnen et al. (Mekonnen et al., 2023)	Ethiopia	Diarrhea	RCT	Hygiene promotion to reduce childhood diarrhea

RCT: randomized controlled trial; HIV: human immunodeficiency virus; STI: sexually transmitted infection; CL: cutaneous leishmaniasis; ARI: acute respiratory infection; PrEP: pre-exposure prophylaxis.

### 3.3 Challenges in conducting research in displaced person settings

**Fig. 2:**
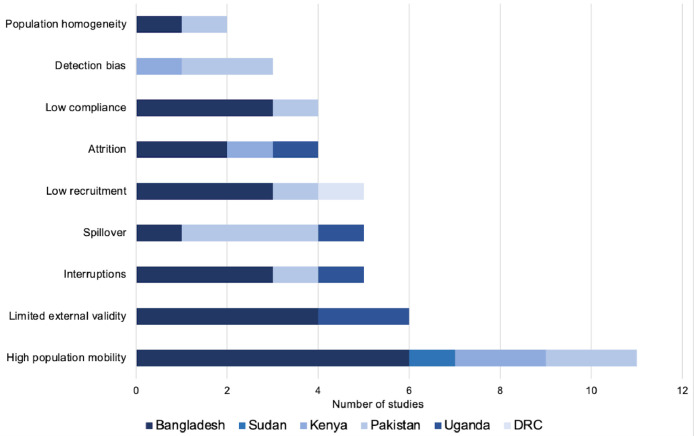
Research challenges in refugee camps and informal settlements by country of study. Studies may have mentioned multiple challenges.

Researchers reported several challenges in conducting research in these settings (Fig. 2). Eleven studies (27.5 %) cited high population mobility as a challenge (Rego et al., 2021; Sanders et al., 2019; Ali et al., 2021; Kaul et al., 2004; Kimani et al., 2006; Kolaczinski et al., 2012; Luby et al., 2005; Luoto et al., 2011; Mitra et al., 1997; Najnin et al., 2019; Pickering et al., 2019), with five studies (12.5 %) requiring that study participants remain in the camp throughout the study’s duration (Rego et al., 2021; Kaul et al., 2004; Kimani et al., 2006; Kolaczinski et al., 2012; Luby et al., 2005). One study reported that 67 % of the baseline population either migrated or died throughout the duration of the study (Ali et al., 2021). Limited external validity (15 %, *n* = 6) (Logie et al., 2023; Ali et al., 2021; Faruque et al., 2021; O’Laughlin et al., 2016; Larson et al., 2010; Pickering et al., 2019), low recruitment (12.5 %, *n* = 5) (ATM Mohiuddin Chowdhury et al., 2022; ATM Mohiuddin Chowdhury et al., 2022; Wolday et al., 1995; Kolaczinski et al., 2012; Pickering et al., 2015), and attrition not specific to population mobility (10 %, *n* = 4) (Stein et al., 2022; Ngure et al., 2022; Rahman et al., 2001; Pickering et al., 2015) were also challenges. Several studies were either interrupted or ended prematurely due to civil unrest (Rego et al., 2021), natural disasters (Rego et al., 2021), COVID-19 (Zohura et al., 2022; Stein et al., 2022), resource constraints (Kolaczinski et al., 2012; Pickering et al., 2019), or operational constraints (Stein et al., 2022).

Camp organization posed several difficulties. The density of the setting led to spillover concerns in five studies (12.5 %) (Ali et al., 2021; Stein et al., 2022; Leslie et al., 2004; Murphy et al., 1996; Owais et al., 2011). One study, however, found that the camp’s structured nature aided in the study’s survey distribution (Sanders et al., 2019). Another study was unable to easily locate households because the official camp map (from the UNHCR) was outdated (Stein et al., 2022). Concerns that the relative homogeneity (specifically, socioeconomic homogeneity) of the population blunted observed associations were expressed in two studies (Brown et al., 2020; Larson et al., 2010). The authors of one study noted that the complex household composition common in their study setting made proper implementation of the intervention difficult (Stein et al., 2022).

In the thirty four studies that tested an intervention, four (12 %) noted low compliance or uptake as a challenge (Brooks et al., 2005; Rowland et al., 1999; Luoto et al., 2011; Najnin et al., 2019). One study credited the low compliance to cultural norms, as the study called for participants not to wash *chaddars* (a cloth used for head covering) that had been treated with insecticide despite norms calling for regular washes (Rowland et al., 1999).

Detection bias was a significant concern noted in several studies (Kaul et al., 2004; Luby et al., 2004; Murphy et al., 1996). This arose over the belief that an intervention’s observed success may not be due to the intervention itself but rather from the increase in overall baseline care, interactions with study staff, regular house visits, and provision of material goods. Lastly, other challenges of note included language barriers (Sanders et al., 2019; Stein et al., 2022) and lack of health or camp records (Sanders et al., 2019; Pickering et al., 2019).

### 3.4 Ethical considerations and community involvement

Of the 33 studies (83 %) that received ethical review board approval, 32 received approval from an ethical review board in the country where the research was performed. Three studies had independent data and safety boards with which researchers met throughout the trial (van der Kop et al., 2018; Larson et al., 2010; Qadri et al., 2003). Only two studies either compensated participants (Kaul et al., 2004) or were cognisant of the potential monetary burden of participating (Kolaczinski et al., 2012). Lastly, 26 studies (65 %) did not give a specific reason for conducting the research in a refugee camp or with displaced people (Mekonnen et al., 2023; Rego et al., 2021; Logie et al., 2023; Zohura et al., 2022; ATM Mohiuddin Chowdhury et al., 2022; Brooks et al., 2005; ATM Mohiuddin Chowdhury et al., 2022; Wolday et al., 1995; van der Kop et al., 2018; Rowland et al., 2001; Kolaczinski et al., 2012; Larson et al., 2010; Luby et al., 2005; Luby et al., 2004; Luoto et al., 2011; Murphy et al., 1996; Ngure et al., 2022; Qadri et al., 2003; Rahman et al., 2001; Rowland et al., 2004; Rowland and Durrani, 1999; Mitra et al., 1997; Najnin et al., 2019; Owais et al., 2011; Pickering et al., 2015; Pickering et al., 2019).

No studies mentioned including the community in the study conception or initial design process. Eighteen studies (45 %) directly involved the community during the study (beyond using local health centers) (Mekonnen et al., 2023; Sanders et al., 2019; Zohura et al., 2022; van der Kop et al., 2018; Stein et al., 2022; Rowland et al., 2001; Rowland et al., 1999; Kaul et al., 2004; Kimani et al., 2006; Kolaczinski et al., 2004; Leslie et al., 2004; Luby et al., 2005; Luby et al., 2004; Rowland et al., 1996; Mitra et al., 1997; Najnin et al., 2019; Owais et al., 2011; Pickering et al., 2019); this was most commonly done via community health workers (CHWs) (25 %, *n* = 10) (van der Kop et al., 2018; Rowland et al., 1999; Kimani et al., 2006; Kolaczinski et al., 2004; Leslie et al., 2004; Luby et al., 2005; Rowland et al., 1996; Mitra et al., 1997; Najnin et al., 2019; Owais et al., 2011) or field workers hired from the community (7.5 %, *n* = 3) (Stein et al., 2022; Rowland et al., 2001; Luby et al., 2004). One study used a translator from the participating camp (Sanders et al., 2019), another used peer educators (Kaul et al., 2004), and a third used local environmental health workers (Mekonnen et al., 2023). Community leaders were involved in three studies by providing informed consent (Luby et al., 2005; Luby et al., 2004) and assisting in community mobilization (Kimani et al., 2006). The community was directly involved in implementing the intervention in two studies; in both studies, participants treated items with insecticide under the study team’s supervision (Rowland et al., 2001; Rowland et al., 1999).

The community was used to inform intervention components in three studies. Importantly, in none of these studies was the community involved at the onset, but rather once the initial conceptualization had already been completed. In one study, the researchers conducted semi-structured interviews with community members to inform intervention modifications (Zohura et al., 2022). Similarly, another study used questionnaire results to inform intervention pricing (Kolaczinski et al., 2004). The community was involved a second time in lowering the price further. In the third study, local ethics experts were consulted to determine an appropriate control (Pickering et al., 2019). However, it is unclear whether ‘local’ refers to community members or if it was being used more broadly.

### 3.5 Intervention feasibility and sustainability

**Fig. 3:**
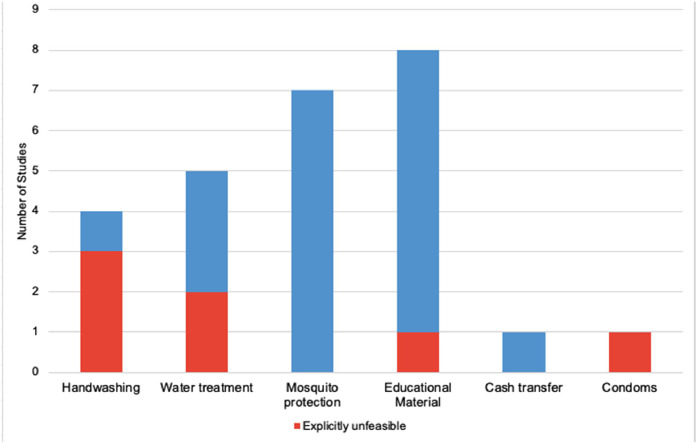
Frequency of intervention types in relevant intervention-based studies included in the review, and the proportion of those studies explicitly considered their intervention unfeasible or unsustainable. Individual studies may have had multiple interventions.

Of the studies that tested an intervention (*n* = 34), 53 % (*n* = 18) provided resources (beyond medical care and/or medication) to participating individuals, households, or communities (Fig. 3) (Mekonnen et al., 2023; Zohura et al., 2022; Stein et al., 2022; Rowland et al., 2001; Rowland et al., 1999; Graham et al., 2002; Kaul et al., 2004; Kimani et al., 2006; Kolaczinski et al., 2004; Luby et al., 2005; Luby et al., 2004; Luoto et al., 2011; Rowland et al., 1996; Rowland et al., 2004; Najnin et al., 2019; Owais et al., 2011; Pickering et al., 2015; Pickering et al., 2019). This included educational materials (24 %, *n* = 8) (Zohura et al., 2022; Stein et al., 2022; Luby et al., 2005; Luby et al., 2004; Luoto et al., 2011; Rowland et al., 2004; Owais et al., 2011; Pickering et al., 2019), bed nets, insecticide, or other mosquito repellents (21 %, *n* = 7) (Rowland et al., 2001; Rowland et al., 1999; Graham et al., 2002; Kimani et al., 2006; Kolaczinski et al., 2004; Rowland et al., 1996; Rowland et al., 2004), water treatment and storage supplies (15 %, *n* = 5) (Zohura et al., 2022; Luoto et al., 2011; Najnin et al., 2019; Pickering et al., 2015; Pickering et al., 2019), hand washing materials (12 %, *n* = 4) (Mekonnen et al., 2023; Zohura et al., 2022; Luby et al., 2005; Najnin et al., 2019), a cash transfer (Stein et al., 2022), and condoms (Kaul et al., 2004). In all but one of these studies, the materials were provided to participants for free and were restocked throughout the study. The one study that imposed a fee for the intervention (insecticide-treated bednets) was purposefully doing so to assess the feasibility of transitioning away from free net distribution and the study planned for free annual retreatment (Kolaczinski et al., 2004). Outside of this, no other studies discussed plans for sustained provision of the intervention(s), even when they were successful. In fact, several studies (24 %, *n* = 8) explicitly noted that the tested interventions (including one clinical intervention) were not feasible or sustainable in the studied location (Mekonnen et al., 2023; Zohura et al., 2022; Kaul et al., 2004; Luby et al., 2005; Luby et al., 2004; Ngure et al., 2022; Pickering et al., 2015; Pickering et al., 2019). There were several reasons for this, but primarily included unavailability of resources and cost (Fig. 3).

In two studies, the materials used for the intervention (a tablet for water treatment and an insecticide) were not available within the studied country (Kimani et al., 2006; Pickering et al., 2015). The tested automated system in the first study was too expensive to be successfully scaled up and affordable in that location (Pickering et al., 2015). Similarly, two handwashing interventions were successful within the studies but were both described as “prohibitively expensive” for large-scale implementation (Luby et al., 2005; Luby et al., 2004). Another study tested a passive water treatment system that required water points to be connected to water storage tanks; only 25 % of water points in the studied area met this standard (Pickering et al., 2019). Two studies noted concerns over whether the successful intervention material would be available once the study team stopped providing it (Mekonnen et al., 2023; Zohura et al., 2022). In one of these studies, the research team noted that the intervention’s effectiveness fell three months after the study ended, perhaps due to “limitations in infrastructure or resources” (Mekonnen et al., 2023). Another study provided “state-of-the-art” health services that were “well above the standard of care for [the] region” and were likely unable to be replicated following the study’s conclusion (Kaul et al., 2004).

Ten studies (29 %) tested pharmacological or clinical interventions (Brooks et al., 2005; Howard et al., 2011; Kolaczinski et al., 2012; Leslie et al., 2004; Murphy et al., 1996; Ngure et al., 2022; Qadri et al., 2003; Rahman et al., 2001; Rowland and Durrani, 1999; Mitra et al., 1997). In three studies (9 %), researchers tested the efficacy of official (at the time) malaria treatment plans (Howard et al., 2011; Kolaczinski et al., 2012; Rowland and Durrani, 1999), two of which had been implemented despite no supporting evidence (Howard et al., 2011; Rowland and Durrani, 1999). The policies were all ineffective; one study's findings were used to significantly alter official malaria treatment guidance (Kolaczinski et al., 2012). Two other studies had the potential for policy implications regarding medication provision, though no related policy changes were discussed in the articles (Leslie et al., 2004; Ngure et al., 2022).

Two interventions had the potential to be cost-effective but were not at the time of study, given large-scale implementation and development issues (Owais et al., 2011; Pickering et al., 2015). Conversely, several (21 %, *n* = 7) studies tested interventions that were already considered cost-effective (Brooks et al., 2005; Rowland et al., 2001; Rowland et al., 1999; Kimani et al., 2006; Ngure et al., 2022; Rowland et al., 1996; Rowland et al., 2004). In four of these studies, the intervention was tested explicitly because a cost-effective measure was needed to replace the status quo (Rowland et al., 2001; Rowland et al., 1999; Kimani et al., 2006; Rowland et al., 1996). Two studies considered cultural norms when choosing an intervention (Rowland et al., 1999; Murphy et al., 1996). In one, researchers tested a homemade wheat-based oral rehydration solution because it allowed women to remain in the home (as is the norm), thereby making it a more accessible solution than typical oral rehydration salts (Murphy et al., 1996). Based on this study, this homemade solution became an approved treatment in the study district (Murphy et al., 1996).

### 3.6 Bias assessments

Across the five criteria for RCTs in the MMAT, 14 of the 29 RCTs (including a crossover RCT) were considered high quality (Mekonnen et al., 2023; Ali et al., 2021; Brooks et al., 2005; van der Kop et al., 2018; Howard et al., 2011; Kaul et al., 2004; Kolaczinski et al., 2012; Larson et al., 2010; Ngure et al., 2022; Qadri et al., 2003; Rahman et al., 2001; Mitra et al., 1997; Owais et al., 2011; Pickering et al., 2019). Both quantitative non-randomized studies were high quality (Brown et al., 2020; Murphy et al., 1996), as were both mixed methods studies (Zohura et al., 2022; Stein et al., 2022) and the single qualitative study (Kolaczinski et al., 2004). Lastly, four of the six quantitative descriptive studies were considered high quality.

The full risk of bias assessment can be found in the **appendix** (Rego et al., 2021; Logie et al., 2023; Sanders et al., 2019; Faruque et al., 2021).

For publication bias, two studies (5 %) had mixed results depending on the age of the participant (Ali et al., 2021; Rowland et al., 1999), twenty-three studies (57 %) reported positive outcomes for their interventions (Mekonnen et al., 2023; Sanders et al., 2019; Zohura et al., 2022; Brooks et al., 2005; Brown et al., 2020; Faruque et al., 2021; Wolday et al., 1995; O’Laughlin et al., 2016; Rowland et al., 2001; Graham et al., 2002; Kimani et al., 2006; Kolaczinski et al., 2004; Kolaczinski et al., 2012; Larson et al., 2010; Luby et al., 2005; Luby et al., 2004; Murphy et al., 1996; Ngure et al., 2022; Qadri et al., 2003; Rahman et al., 2001; Rowland et al., 1996; Owais et al., 2011; Pickering et al., 2019), and 15 (37.5 %) reported negative outcomes (Rego et al., 2021; Logie et al., 2023; ATM Mohiuddin Chowdhury et al., 2022; ATM Mohiuddin Chowdhury et al., 2022; van der Kop et al., 2018; Stein et al., 2022; Howard et al., 2011; Kaul et al., 2004; Leslie et al., 2004; Luoto et al., 2011; Rowland et al., 2004; Rowland and Durrani, 1999; Mitra et al., 1997; Najnin et al., 2019; Pickering et al., 2015). Among the 40 included studies, one individual (M. Rowland) was either the first or senior author on ten studies (Rowland et al., 2001; Rowland et al., 1999; Graham et al., 2002; Howard et al., 2011; Kolaczinski et al., 2004; Kolaczinski et al., 2012; Leslie et al., 2004; Rowland et al., 1996; Rowland et al., 2004; Rowland and Durrani, 1999). Another author (S. Luby) was either the first or senior author on five studies, meaning that two authors were responsible for nearly 40 % of the studies included in this review (Luby et al., 2005; Luby et al., 2004; Najnin et al., 2019; Pickering et al., 2015; Pickering et al., 2019).

## 4 Discussion

To our knowledge, this review is the first to assess the landscape of infectious disease research conducted in refugee camps and informal settlements in LMICs. Despite existing guidelines for conducting research with refugees or other contexts of displacement that emphasize community participation and intervention sustainability, we found that most studies fell short across several metrics. None of the included studies involved the participating community in the study conception or design process. Fig. 3 illustrates that in 20 % of studies, researchers tested interventions that they explicitly noted were not feasible to implement in the studied context. Such findings are stark reminders that there is still significant room for improvement in the ethical conduct of research in these settings.

Several studies noted particular difficulties with conducting research in refugee camps and informal settlements, many of which are likely inherent to these settings, such as high population mobility. Such realities are perhaps foreseeable in these settings, though there was little discussion of how the research was adapted to fit them. Instead, many studies required that participants alter their behaviors for the study: for example, several studies required that participants stay in the camp throughout the duration. Such requirements highlight tensions between the realities of camp settings, unique population dynamics, and rigid research methodologies that must be addressed both explicitly and under ethical guidance. Similarly, research within a camp (and especially that which involves an intervention) inherently changes camp dynamics through the introduction of resources, attention, and funding. However, only three studies noted concerns over detection bias, despite many of the intervention-based studies providing resources that may otherwise be difficult to obtain for the community. Overwhelmingly, studies in this review lacked a critical discussion of how the research’s end may impact the community, especially when the conclusion of the research corresponds to a concurrent conclusion in resource availability.

There was also a noticeable imbalance between countries in which research was conducted. Despite Iran hosting the most refugees for an LMIC, there were no Iran-based studies that met our inclusion criteria. Pakistan and Bangladesh were overrepresented in the included studies (Tables 1 and 2). Notably, most of the studies in Bangladesh involved the International Center for Diarrheal Disease Research, Bangladesh (icddr,b), indicating that there is strong institutional support for related research in Bangladesh. This may also explain the heavy focus on diarrheal disease in the Bangladesh-based research. The included literature was heavily weighted toward diarrhea and malaria research, though similar to the concentration of diarrheal research in Bangladesh, all but three studies on malaria were conducted in Pakistan. While diarrheal diseases and malaria are undoubtedly worthwhile focuses of research in these settings, there are other infectious diseases, such as leishmaniasis or skin infections, that are particularly relevant in displaced populations and receive little attention.

Less than half of the studies in this review involved the community during the study period, and the majority of this involvement was through CHWs. The benefits of using CHWs have been well-documented in the literature, and such inclusion is important (Landers and Levinson, 2016). However, there were only three studies that explicitly discussed researchers being informed *by* the community. Such iterative collaboration on the research process is essential for the co-construction of research and likewise may aid in the development of projects that meet the community’s highest perceived needs.

This review has several limitations. Due to the limited amount of databases searched, there is a possibility of additional, relevant literature that was not included within our study. Furthermore, it is possible that we missed studies that meet the inclusion criteria due to different or changing terminology. We attempted to mitigate this through the use of comprehensive search terms and checking these terms with relevant experts. It is also possible that we misclassified some studies because the authors did not explicitly state the information in the text. For example, a study may have included community members in their research but did not explicitly state this in the final article, and we, therefore, did not count it as involving the community. We limited our data extraction to explicit mentions, however, to avoid subjective assumptions by our research team. We also viewed each of these studies in isolation, which may mask researchers’ prolonged involvement with certain communities. The articles in this review span nearly 30 years, during which time ethical standards of research, and indeed research practice generally, have evolved. However, we applied the same rigorous ethical expectations across each study, as the lack of formal guidelines should not be used to excuse unethical research practices.

Our findings highlight a critical and an unmet need for practical guidelines on conducting ethically sound research with displaced communities, both in LMICs and elsewhere, that respond to challenges experienced by researchers. Though other guidelines exist, there is a disconnect with implementing them in these complex settings; this review helps indicate where these gaps between framework ideals and practical implementation often reside. The forced migration experience is unique and dynamic, and more research must be attuned to the specific needs affected communities face. Moreover, more work must be done by researchers to critically assess their research, namely to critique the necessity of the research (who does it benefit?), the appropriateness of the intervention (is this intervention accessible and sustainable in this context?), the frequency of collaboration with the community (has the community led this work?), and the effects of the work’s presence after the project is completed (how will this change the community?). These deliberations should be prominent in the published work from the project and, indeed, should be expected by journal editors and readers.

## 5 Conclusion

In conclusion, research conducted in refugee camps and informal settlements in LMICs frequently falls short in several areas, including involving the community throughout the research process and implementing appropriate interventions (especially when considering long-term feasibility). That no studies explicitly mentioned including the community in study conception or design is particularly concerning. We also find that research in these settings presents unique challenges, many of which may not be addressed in existing research frameworks or guidelines. Such work may require methodological adjustments in response to this dynamic and complex context, which should be better understood throughout the research process and by publishers and funders, which may allow researchers more latitude to make these necessary adjustments. Lastly, there is a disconnect between where displaced individuals reside and where research is done, both at the country level and between high-income countries and LMICs. It is critical that the unique health challenges faced by displaced individuals in these contexts are better understood, especially given the external challenges and marginalization faced by displaced communities. Reestablishing the health and well-being of participating displaced communities as the core priority of health research can be aided by more conscious efforts to conduct ethical research that is informed by - and adjusted to - the community itself, including through further research in frameworks that can guide researchers.

## Funding

The work was supported by a Wellcome Trust contract to 10.13039/100007161Boston University (Contract Number C-010656).

## Data sharing

All data used for this study has been included in the manuscript or supplementary material.

## CRediT authorship contribution statement

**Neila Gross:** Writing – review & editing, Writing – original draft, Methodology, Investigation, Formal analysis. **Maia C. Tarnas:** Writing – review & editing, Writing – original draft, Methodology, Investigation, Formal analysis. **Rashmina J. Sayeeda:** Writing – review & editing, Methodology, Investigation. **Carly Ching:** Writing – review & editing, Conceptualization. **David Flynn:** Methodology. **Muhammad H Zaman:** Writing – review & editing, Funding acquisition, Conceptualization.

## Declaration of competing interest

The authors declare that they have no known competing financial interests or personal relationships that could have appeared to influence the work reported in this paper.

## Appendix Supplementary materials

Image, application 1
