# Supplementary material for: Infectious disease research in forcibly displaced populations: A systematic review in low- and middle-income host countries
Source: J Migr Health. 2025 Jun 27;12:100341. doi: 10.1016/j.jmh.2025.100341 (PMC12271074; doi:10.1016/j.jmh.2025.100341)
Supplement: Supplementary file 1 [file mmc1.docx]

Supplementary Material

**Table of Contents**

[**Acronyms and Abbreviations** 2](#_Toc155965729)

[[**Preferred Reporting Items for Systematic Reviews and Meta-Analyses (PRISMA) Compliance**](#_tfquc4ff5237) 3](#_Toc155965568)

[**Search Strategy** 8](#_Toc155965731)

[**Bias Assessments** 16](#_Toc155965732)

[*Publication Bias* 16](#_Toc155965733)

[*Study Risk of Bias* 16](#_Toc155965734)

[**Post Abstract Screening Cuts** 21](#_Toc155965735)

[**References** 24](#_Toc155965736)

# **Acronyms and Abbreviations**

UNHCR- United Nations Refugee Agency or United Nations High Commissioner for Refugees

LMICs- Low- and Middle-Income Countries

WASH- Water, Sanitation and Hygiene

WHO- World Health Organization

UNICEF- United Nations Children’s Fund

DRC- Democratic Republic of the Congo

MMAT- Mixed Methods Appraisal Tool

CL- cutaneous leishmaniasis

STI- sexually transmitted infection

RCT-randomized control trial

ARI-acute respiratory infection

Hep B- Hepatitis B

DTP-Diphtheria Tetanus and Pertussis

CHW- Community Health Worker

ICDDR,B - International Center for Diarrheal Disease Research, Bangladesh

# **PRISMA Compliance**

This study complies with the [PRISMA 2020 recommendations](http://www.prisma-statement.org/). The table below shows the completed checklist and the locations in which the respective items can be found within the paper.

| **Section and Topic** | **Item #** | **Checklist item** | **Location where item is reported** |
| --- | --- | --- | --- |
| **TITLE** | | |  |
| Title | 1 | Identify the report as a systematic review. | Paper title |
| **ABSTRACT** | | |  |
| Abstract | 2 | See the PRISMA 2020 for Abstracts checklist. | N/A |
| **INTRODUCTION** | | |  |
| Rationale | 3 | Describe the rationale for the review in the context of existing knowledge. | Main text introduction, pg.1 |
| Objectives | 4 | Provide an explicit statement of the objective(s) or question(s) the review addresses. | Main text introduction, pg.1 final sentence. |
| **METHODS** | | |  |
| Eligibility criteria | 5 | Specify the inclusion and exclusion criteria for the review and how studies were grouped for the syntheses. | Main text methods, second paragraph, pg. 2 |
| Information sources | 6 | Specify all databases, registers, websites, organisations, reference lists and other sources searched or consulted to identify studies. Specify the date when each source was last searched or consulted. | Main text methods, second paragraph, pg. 2 |
| Search strategy | 7 | Present the full search strategies for all databases, registers and websites, including any filters and limits used. | Supplementary Material, pg. 8 |
| Selection process | 8 | Specify the methods used to decide whether a study met the inclusion criteria of the review, including how many reviewers screened each record and each report retrieved, whether they worked independently, and if applicable, details of automation tools used in the process. | Main text methods, second paragraph, pg. 2 |
| Data collection process | 9 | Specify the methods used to collect data from reports, including how many reviewers collected data from each report, whether they worked independently, any processes for obtaining or confirming data from study investigators, and if applicable, details of automation tools used in the process. | Main text methods, third paragraph, pg. 2 |
| Data items | 10a | List and define all outcomes for which data were sought. Specify whether all results that were compatible with each outcome domain in each study were sought (e.g. for all measures, time points, analyses), and if not, the methods used to decide which results to collect. | Main text methods, third paragraph, pg. 2 |
|  | 10b | List and define all other variables for which data were sought (e.g. participant and intervention characteristics, funding sources). Describe any assumptions made about any missing or unclear information. | Main text methods, third paragraph, pg. 2 |
| Study risk of bias assessment | 11 | Specify the methods used to assess risk of bias in the included studies, including details of the tool(s) used, how many reviewers assessed each study and whether they worked independently, and if applicable, details of automation tools used in the process. | Supplementary Material, “Risk of bias” section, pg. 16 |
| Effect measures | 12 | Specify for each outcome the effect measure(s) (e.g. risk ratio, mean difference) used in the synthesis or presentation of results. | N/A |
| Synthesis methods | 13a | Describe the processes used to decide which studies were eligible for each synthesis (e.g. tabulating the study intervention characteristics and comparing against the planned groups for each synthesis (item #5)). | N/A |
|  | 13b | Describe any methods required to prepare the data for presentation or synthesis, such as handling of missing summary statistics, or data conversions. | N/A |
|  | 13c | Describe any methods used to tabulate or visually display results of individual studies and syntheses. | Main text methods, “Data Extraction and analysis,” pg. 2 |
|  | 13d | Describe any methods used to synthesize results and provide a rationale for the choice(s). If meta-analysis was performed, describe the model(s), method(s) to identify the presence and extent of statistical heterogeneity, and software package(s) used. | Main text methods, “Data Extraction and analysis,” pg. 2 |
|  | 13e | Describe any methods used to explore possible causes of heterogeneity among study results (e.g. subgroup analysis, meta-regression). | Main text methods, “Data Extraction and analysis,” pg. 2 |
|  | 13f | Describe any sensitivity analyses conducted to assess robustness of the synthesized results. | Main text methods, “Data Extraction and analysis,” pg. 2 |
| Reporting bias assessment | 14 | Describe any methods used to assess risk of bias due to missing results in a synthesis (arising from reporting biases). | Supplementary Material, “Risk of Bias,” Page 16-20 |
| Certainty assessment | 15 | Describe any methods used to assess certainty (or confidence) in the body of evidence for an outcome. | N/A |
| **RESULTS** | | |  |
| Study selection | 16a | Describe the results of the search and selection process, from the number of records identified in the search to the number of studies included in the review, ideally using a flow diagram. | Main text Results, “Screening Results,” pg. 3 |
|  | 16b | Cite studies that might appear to meet the inclusion criteria, but which were excluded, and explain why they were excluded. | Supplementary Material, “Excluded Study Characteristics,” pg. 21-23 |
| Study characteristics | 17 | Cite each included study and present its characteristics. | Main text Results, “Study Characteristics,” pg. 3-5 |
| Risk of bias in studies | 18 | Present assessments of risk of bias for each included study. | Supplementary Material, “Risk of Bias,” pg. 16-20 |
| Results of individual studies | 19 | For all outcomes, present, for each study: (a) summary statistics for each group (where appropriate) and (b) an effect estimate and its precision (e.g. confidence/credible interval), ideally using structured tables or plots. | Main text Results, “Screening Results” Table 1, pg. 3-5 |
| Results of syntheses | 20a | For each synthesis, briefly summarise the characteristics and risk of bias among contributing studies. | Supplementary Material, “Risk of Bias”, pg. 16 |
|  | 20b | Present results of all statistical syntheses conducted. If meta-analysis was done, present for each the summary estimate and its precision (e.g. confidence/credible interval) and measures of statistical heterogeneity. If comparing groups, describe the direction of the effect. | N/A |
|  | 20c | Present results of all investigations of possible causes of heterogeneity among study results. | N/A |
|  | 20d | Present results of all sensitivity analyses conducted to assess the robustness of the synthesized results. | N/A |
| Reporting biases | 21 | Present assessments of risk of bias due to missing results (arising from reporting biases) for each synthesis assessed. | Supplementary Material, “Risk of Bias,” pg. 16-20 |
| Certainty of evidence | 22 | Present assessments of certainty (or confidence) in the body of evidence for each outcome assessed. | N/A |
| **DISCUSSION** | | |  |
| Discussion | 23a | Provide a general interpretation of the results in the context of other evidence. | Main text Discussion, pg. 10-12 |
|  | 23b | Discuss any limitations of the evidence included in the review. | Main text Discussion pg. 11 |
|  | 23c | Discuss any limitations of the review processes used. | Main text Discussion pg. 11 |
|  | 23d | Discuss implications of the results for practice, policy, and future research. | Main text Discussion pg. 11 |
| **OTHER INFORMATION** | | |  |
| Registration and protocol | 24a | Provide registration information for the review, including register name and registration number, or state that the review was not registered. | Main Text Methods pg. 2 |
|  | 24b | Indicate where the review protocol can be accessed, or state that a protocol was not prepared. | Main text Methods pg. 2 |
|  | 24c | Describe and explain any amendments to information provided at registration or in the protocol. | Supplementary Material, “Search Strategy”, pg. 8-15 |
| Support | 25 | Describe sources of financial or non-financial support for the review, and the role of the funders or sponsors in the review. | Main text Methods, “Role of the Funding Source,” pg. 3 |
| Competing interests | 26 | Declare any competing interests of review authors. | Main text, “Competing interests,” pg. 12 |
| Availability of data, code and other materials | 27 | Report which of the following are publicly available and where they can be found: template data collection forms; data extracted from included studies; data used for all analyses; analytic code; any other materials used in the review. | Main text, “Data Sharing,” pg. 13 |

# **Search Strategy**

*Literature Search*

We used electronic bibliographic databases for peer-reviewed publications that were searched using a list of MeSH terms and compiled through Rayyan. Our original protocol included a gray literature search, which will be included in a subsequent study. The rest of the protocol was followed throughout the review process.

*Electronic Bibliographic Databases*

The following electronic bibliographic databases were searched: PubMed, Embase, and Web of Science (WoS). We systematically searched using MeSH terms and free-text keywords to ensure comprehensive retrieval across databases. The search strategy was constructed using controlled vocabulary from PubMed’s MeSH database—compiled using Boston University’s MeSH Miner^1^—and refined with free-text terms to capture relevant articles in Embase and Web of Science, which do not consistently use MeSH indexing. The following terms were elucidated from the described methods above to reflect any term that was associated with (1) infectious/communicable diseases, (2) refugee populations, and (3) the top ten low to middle-income host countries defined by the UNHCR.[^2^](https://www.zotero.org/google-docs/?e6ar5b)

(((((("Refugees"[Mesh] OR "Refugee"[tw] OR "Political Asylum Seekers"[tw] OR "Asylum Seeker, Political"[tw] OR "Asylum Seekers, Political"[tw] OR "Political Asylum Seeker"[tw] OR "Seekers, Political Asylum"[tw] OR "Political Refugees"[tw] OR "Political Refugee"[tw] OR "Refugee, Political"[tw] OR "Refugees, Political"[tw] OR "Asylum Seekers"[tw] OR "Asylum Seeker"[tw] OR "Seeker, Asylum"[tw] OR "Seekers, Asylum"[tw] OR "Displaced Persons"[tw] OR "Displaced Person"[tw] OR "Person, Displaced"[tw] OR "Persons, Displaced"[tw] OR "Internally Displaced Persons"[tw] OR "Displaced Person, Internally"[tw] OR "Displaced Persons, Internally"[tw] OR "Internally Displaced Person"[tw]) OR ("Refugee Camps"[Mesh] OR "Camp, Refugee"[tw] OR "Camps, Refugee"[tw] OR "Refugee Camp"[tw])) OR ("Poverty"[Mesh] OR "Extreme Poverty"[tw] OR "Poverty, Extreme"[tw] OR "Absolute Poverty"[tw] OR "Poverty, Absolute"[tw] OR "Indigents"[tw] OR "Indigent"[tw] OR "Indigency"[tw] OR "Federal Poverty Threshold"[tw] OR "Poverty Threshold, Federal"[tw] OR "Federal Poverty Level"[tw] OR "Federal Poverty Levels"[tw] OR "Level, Federal Poverty"[tw] OR "Poverty Level, Federal"[tw] OR "Low-Income Population"[tw] OR "Low-Income Populations"[tw] OR "Population, Low-Income"[tw] OR "Low Income Population"[tw] OR "Low Income Populations"[tw] OR "Population, Low Income"[tw])) OR ("Poverty Areas"[Mesh] OR "Area, Poverty"[tw] OR "Areas, Poverty"[tw] OR "Poverty Area"[tw] OR "Slums"[tw] OR "Ghettos"[tw] OR "Ghetto"[tw])) OR ("Refugees"[Mesh] OR "Refugee"[tw] OR "Political "[tw] OR " Seekers"[tw] OR " Seeker, Political"[tw] OR " Seekers, Political"[tw] OR "Political "[tw] OR " Seeker"[tw] OR "Seekers, Political "[tw] OR "Political Refugees"[tw] OR "Political Refugee"[tw] OR "Refugee, Political"[tw] OR "Refugees, Political"[tw] OR " Seekers"[tw] OR " Seeker"[tw] OR "Seeker, "[tw] OR "Seekers, "[tw] OR "Displaced Persons"[tw] OR "Displaced Person"[tw] OR "Person, Displaced"[tw] OR "Persons, Displaced"[tw] OR "Internally Displaced Persons"[tw] OR "Displaced Person, Internally"[tw] OR "Displaced Persons, Internally"[tw] OR "Internally Displaced Person"[tw]) OR ("Medically Underserved Area"[Mesh] OR "Areas, Medically Underserved"[tw] OR "Medically Underserved Areas"[tw] OR "Underserved Area, Medically"[tw] OR "Underserved Areas, Medically"[tw] OR "Area, Medically Underserved"[tw] OR "Medically Underserved Population"[tw] OR "Medically Underserved Populations"[tw] OR "Population, Medically Underserved"[tw] OR "Populations, Medically Underserved"[tw] OR "Underserved Population, Medically"[tw] OR "Underserved Populations, Medically"[tw] OR "Physician Shortage Area"[tw] OR "Area, Physician Shortage"[tw] OR "Areas, Physician Shortage"[tw] OR "Physician Shortage Areas"[tw] OR "Shortage Area, Physician"[tw] OR "Shortage Areas, Physician"[tw] OR "National "[tw] OR " Corps"[tw] OR " Corps, National"[tw])) AND ((((((((((((((((((((((((((((((((((((((((((((((((("Disease"[Mesh] OR "Diseases"[tw]) OR ("Early Diagnosis"[Mesh] OR "Diagnosis, Early"[tw] OR "Early Detection of Disease"[tw] OR "Disease Early Detection"[tw])) OR ("Disease Hotspot"[Mesh] OR "Disease Hotspots"[tw] OR "Hotspot, Disease"[tw] OR "Hotspots, Disease"[tw] OR "Disease Hotspot Burden"[tw] OR "Burden, Disease Hotspot"[tw] OR "Burdens, Disease Hotspot"[tw] OR "Disease Hotspot Burdens"[tw] OR "Hotspot Burden, Disease"[tw] OR "Hotspot Burdens, Disease"[tw] OR "Spatial Cluster, Disease"[tw] OR "Cluster, Disease Spatial"[tw] OR "Clusters, Disease Spatial"[tw] OR "Disease Spatial Cluster"[tw] OR "Disease Spatial Clusters"[tw] OR "Spatial Clusters, Disease"[tw] OR "Transmission Hotspot"[tw] OR "Hotspot, Transmission"[tw] OR "Hotspots, Transmission"[tw] OR "Transmission Hotspots"[tw] OR "Burden Hotspot"[tw] OR "Burden Hotspots"[tw] OR "Hotspot, Burden"[tw] OR "Hotspots, Burden"[tw] OR "Disease Clusters"[tw] OR "Cluster, Disease"[tw] OR "Clusters, Disease"[tw] OR "Disease Cluster"[tw] OR "Disease Hot Spot"[tw] OR "Disease Hot Spots"[tw] OR "Hot Spot, Disease"[tw] OR "Hot Spots, Disease"[tw] OR "Disease Clustering"[tw] OR "Clustering, Disease"[tw] OR "Clusterings, Disease"[tw] OR "Disease Clusterings"[tw])) OR ("Infectious Disease Transmission, Vertical"[Mesh] OR "Pathogen Transmission, Vertical"[tw] OR "Transmission, Vertical Pathogen"[tw] OR "Vertical Pathogen Transmission"[tw] OR "Vertical Transmission of Infectious Disease"[tw] OR "Vertical Infection Transmission"[tw] OR "Vertical Infectious Disease Transmission"[tw] OR "Infection Transmission, Vertical"[tw] OR "Transmission, Vertical Infection"[tw] OR "Maternal-Fetal Infection Transmission"[tw] OR "Maternal Fetal Infection Transmission"[tw] OR "Transmission, Maternal-Fetal Infection"[tw] OR "Infection Transmission, Maternal-Fetal"[tw] OR "Infection Transmission, Maternal Fetal"[tw] OR "Infection Transmission, Fetomaternal"[tw] OR "Mother-to-Child Transmission"[tw] OR "Mother to Child Transmission"[tw] OR "Mother-to-Child Transmissions"[tw] OR "Transmission, Mother-to-Child"[tw] OR "Transmissions, Mother-to-Child"[tw] OR "Fetomaternal Infection Transmission"[tw] OR "Transmission, Fetomaternal Infection"[tw])) OR ("Communicable Diseases, Emerging"[Mesh] OR "Communicable Disease, Emerging"[tw] OR "Disease, Emerging Communicable"[tw] OR "Diseases, Emerging Communicable"[tw] OR "Emerging Communicable Disease"[tw] OR "Emerging Communicable Diseases"[tw] OR "Infectious Diseases, Emerging"[tw] OR "Disease, Emerging Infectious"[tw] OR "Diseases, Emerging Infectious"[tw] OR "Emerging Infectious Disease"[tw] OR "Emerging Infectious Diseases"[tw] OR "Infectious Disease, Emerging"[tw] OR "Communicable Diseases, Reemerging"[tw] OR "Communicable Disease, Reemerging"[tw] OR "Disease, Reemerging Communicable"[tw] OR "Diseases, Reemerging Communicable"[tw] OR "Reemerging Communicable Disease"[tw] OR "Reemerging Communicable Diseases"[tw] OR "Communicable Diseases, Re-Emerging"[tw] OR "Communicable Disease, Re-Emerging"[tw] OR "Communicable Diseases, Re Emerging"[tw] OR "Disease, Re-Emerging Communicable"[tw] OR "Diseases, Re-Emerging Communicable"[tw] OR "Re-Emerging Communicable Disease"[tw] OR "Re-Emerging Communicable Diseases"[tw] OR "Infectious Diseases, Re-Emerging"[tw] OR "Disease, Re-Emerging Infectious"[tw] OR "Diseases, Re-Emerging Infectious"[tw] OR "Infectious Disease, Re-Emerging"[tw] OR "Infectious Diseases, Re Emerging"[tw] OR "Re-Emerging Infectious Disease"[tw] OR "Re-Emerging Infectious Diseases"[tw] OR "Infectious Diseases, Reemerging"[tw] OR "Disease, Reemerging Infectious"[tw] OR "Diseases, Reemerging Infectious"[tw] OR "Infectious Disease, Reemerging"[tw] OR "Reemerging Infectious Disease"[tw] OR "Reemerging Infectious Diseases"[tw])) OR ("Disease Progression"[Mesh] OR "Progression, Disease"[tw] OR "Clinical Course"[tw] OR "Clinical Progression"[tw] OR "Progression, Clinical"[tw] OR "Disease Exacerbation"[tw] OR "Exacerbation, Disease"[tw])) OR ("Communicable Disease Control"[Mesh] OR "Control, Communicable Disease"[tw] OR "Parasite Control"[tw] OR "Control, Parasite"[tw] OR "Flattening the Curve, Communicable Disease Control"[tw] OR "Flatten the Curve of Epidemic"[tw])) OR ("Secondary Prevention"[Mesh] OR "Prevention, Secondary"[tw] OR "Preventions, Secondary"[tw] OR "Secondary Preventions"[tw] OR "Secondary Disease Prevention"[tw] OR "Disease Prevention, Secondary"[tw] OR "Disease Preventions, Secondary"[tw] OR "Prevention, Secondary Disease"[tw] OR "Preventions, Secondary Disease"[tw] OR "Secondary Disease Preventions"[tw] OR "Relapse Prevention"[tw] OR "Prevention, Relapse"[tw] OR "Preventions, Relapse"[tw] OR "Relapse Preventions"[tw] OR "Early Therapy"[tw] OR "Early Therapies"[tw] OR "Therapies, Early"[tw] OR "Therapy, Early"[tw])) OR ("Primary Prevention"[Mesh] OR "Disease Prevention, Primary"[tw] OR "Disease Preventions, Primary"[tw] OR "Primary Disease Prevention"[tw] OR "Primary Disease Preventions"[tw] OR "Prevention, Primary"[tw] OR "Primordial Prevention"[tw] OR "Preventions, Primordial"[tw] OR "Primordial Preventions"[tw] OR "Prevention, Primordial"[tw])) OR ("Point-of-Care Testing"[Mesh] OR "Testing, Point-of-Care"[tw] OR "Point-Of-Care Diagnostic Testing"[tw] OR "Diagnostic Testing, Point-Of-Care"[tw] OR "Point Of Care Diagnostic Testing"[tw] OR "Testing, Point-Of-Care Diagnostic"[tw] OR "Point-Of-Care Diagnostic Tests"[tw] OR "Diagnostic Test, Point-Of-Care"[tw] OR "Diagnostic Tests, Point-Of-Care"[tw] OR "Point Of Care Diagnostic Tests"[tw] OR "Point-Of-Care Diagnostic Test"[tw] OR "Test, Point-Of-Care Diagnostic"[tw] OR "Tests, Point-Of-Care Diagnostic"[tw] OR "Point-Of-Care Tests"[tw] OR "Point Of Care Tests"[tw] OR "Point-Of-Care Test"[tw] OR "Point Of Care Test"[tw] OR "Test, Point-Of-Care"[tw] OR "Tests, Point-Of-Care"[tw] OR "Point of Care Testing"[tw] OR "Point-Of-Care Diagnostics"[tw] OR "Diagnostic, Point-Of-Care"[tw] OR "Diagnostics, Point-Of-Care"[tw] OR "Point Of Care Diagnostics"[tw] OR "Point-Of-Care Diagnostic"[tw] OR "Bedside Testing"[tw] OR "Testing, Bedside"[tw])) OR ("Point-of-Care Systems"[Mesh] OR "Point of Care Systems"[tw] OR "Point-of-Care System"[tw] OR "Systems, Point-of-Care"[tw] OR "Point-of-Care"[tw] OR "Point of Care"[tw] OR "Bedside Computing"[tw] OR "Computing, Bedside"[tw] OR "Point of Care Technology"[tw] OR "Bedside Technology"[tw] OR "Bedside Technologies"[tw] OR "Technologies, Bedside"[tw] OR "Technology, Bedside"[tw])) OR ("Disease Resistance"[Mesh] OR "Disease Resistances"[tw] OR "Resistances, Disease"[tw] OR "Resistance, Disease"[tw])) OR ("Infectious Disease Medicine"[Mesh] OR "Disease Medicine, Infectious"[tw] OR "Medicine, Infectious Disease"[tw] OR "Infectious Diseases Specialty"[tw] OR "Diseases Specialty, Infectious"[tw] OR "Infectious Diseases Specialties"[tw] OR "Specialties, Infectious Diseases"[tw] OR "Specialty, Infectious Diseases"[tw] OR "Infectious Disease Specialty"[tw] OR "Infectious Disease Specialties"[tw] OR "Specialties, Infectious Disease"[tw] OR "Specialty, Infectious Disease"[tw])) OR ("Therapeutics"[Mesh] OR "Therapeutic"[tw] OR "Therapy"[tw] OR "Therapies"[tw] OR "Treatment"[tw] OR "Treatments"[tw])) OR ("therapy [Subheading]"[Mesh] OR "treatment"[tw] OR "disease management"[tw])) OR ("Time-to-Treatment"[Mesh] OR "Time-to-Treatments"[tw] OR "Time to Treatment"[tw] OR "Time to Treatments"[tw] OR "Door-to-Treatment Time"[tw] OR "Door to Treatment Time"[tw] OR "Delayed Treatment"[tw] OR "Delayed Treatments"[tw] OR "Treatment, Delayed"[tw] OR "Treatment Delay"[tw] OR "Delay, Treatment"[tw] OR "Treatment Delays"[tw])) OR ("Treatment Failure"[Mesh] OR "Failure, Treatment"[tw] OR "Failures, Treatment"[tw] OR "Treatment Failures"[tw])) OR ("Treatment Outcome"[Mesh] OR "Outcome, Treatment"[tw] OR "Patient-Relevant Outcome"[tw] OR "Outcome, Patient-Relevant"[tw] OR "Outcomes, Patient-Relevant"[tw] OR "Patient Relevant Outcome"[tw] OR "Patient-Relevant Outcomes"[tw] OR "Clinical Effectiveness"[tw] OR "Effectiveness, Clinical"[tw] OR "Treatment Effectiveness"[tw] OR "Effectiveness, Treatment"[tw] OR "Rehabilitation Outcome"[tw] OR "Outcome, Rehabilitation"[tw] OR "Treatment Efficacy"[tw] OR "Efficacy, Treatment"[tw] OR "Clinical Efficacy"[tw] OR "Efficacy, Clinical"[tw])) OR ("Treatment Refusal"[Mesh] OR "Refusal, Treatment"[tw] OR "Refusals, Treatment"[tw] OR "Treatment Refusals"[tw] OR "Patient Refusal of Treatment"[tw] OR "Refusal of Treatment"[tw] OR "Anesthesia Refusal"[tw] OR "Anesthesia Refusals"[tw] OR "Refusal, Anesthesia"[tw] OR "Refusals, Anesthesia"[tw] OR "Patient Elopement"[tw] OR "Elopement, Patient"[tw] OR "Elopements, Patient"[tw] OR "Patient Elopements"[tw])) OR ("Aftercare"[Mesh] OR "After Care"[tw] OR "After-Treatment"[tw] OR "After Treatment"[tw] OR "After-Treatments"[tw] OR "Follow-Up Care"[tw] OR "Care, Follow-Up"[tw] OR "Cares, Follow-Up"[tw] OR "Follow Up Care"[tw] OR "Follow-Up Cares"[tw] OR "Postabortion"[tw] OR "Postabortal Programs"[tw] OR "Postabortal Program"[tw] OR "Program, Postabortal"[tw] OR "Programs, Postabortal"[tw])) OR ("Health Policy"[Mesh] OR "Policies, Health"[tw] OR "Policy, Health"[tw] OR "Healthcare Policy"[tw] OR "Healthcare Policies"[tw] OR "Policies, Healthcare"[tw] OR "Policy, Healthcare"[tw] OR "Health Policies"[tw] OR "Health Care Policies"[tw] OR "Care Policies, Health"[tw] OR "Health Care Policy"[tw] OR "Policies, Health Care"[tw] OR "Policy, Health Care"[tw] OR "National Health Policy"[tw] OR "Health Policy, National"[tw] OR "National Health Policies"[tw])) OR ("Public Reporting of Healthcare Data"[Mesh] OR "Quality Data Reporting"[tw] OR "Data Reporting, Quality"[tw] OR "Reporting, Quality Data"[tw] OR "Public Reporting of Health Care Data"[tw] OR "Mandated Quality Data Reporting"[tw] OR "Public Reporting of Quality Data"[tw])) OR ("Routinely Collected Health Data"[Mesh] OR "Routinely-Collected Health Data"[tw] OR "Health Data, Routinely-Collected"[tw] OR "Wearable Device Data"[tw] OR "Data, Wearable Device"[tw] OR "Device Data, Wearable"[tw] OR "Registry Data"[tw] OR "Data, Registry"[tw] OR "Routinely Collected Data"[tw] OR "Routinely-Collected Data"[tw] OR "Data, Routinely-Collected"[tw] OR "Health Administrative Data"[tw] OR "Administrative Data, Health"[tw] OR "Data, Health Administrative"[tw] OR "Health Administrative Datas"[tw])) OR ("Public Health Surveillance"[Mesh] OR "Surveillance, Public Health"[tw])) OR ("Benchmarking"[Mesh] OR "Best Practice Analysis"[tw] OR "Analysis, Best Practice"[tw] OR "Benchmarks"[tw] OR "Metrics"[tw] OR "Benchmark"[tw] OR ", Health Care"[tw] OR "Health Care "[tw] OR ", Healthcare"[tw])) OR ("Right to Health"[Mesh] OR "Right to Health Care"[tw] OR "Right to Healthcare"[tw] OR "Healthcare, Right to"[tw] OR "Healthcares, Right to"[tw] OR "Health Rights"[tw] OR "Healthcare Rights"[tw] OR "Healthcare Right"[tw] OR "Right, Healthcare"[tw] OR "Rights, Healthcare"[tw])) OR ("Disease Notification"[Mesh] OR "Infectious Disease Reporting"[tw] OR "Disease Reporting, Infectious"[tw] OR "Disease Reportings, Infectious"[tw] OR "Infectious Disease Reportings"[tw] OR "Reporting, Infectious Disease"[tw] OR "Reportings, Infectious Disease"[tw] OR "Notification, Disease"[tw] OR "Disease Notifications"[tw] OR "Notifications, Disease"[tw] OR "Exposure Notification"[tw] OR "Exposure Notifications"[tw] OR "Notification, Exposure"[tw] OR "Notifications, Exposure"[tw])) OR ("Evidence-Based Practice"[Mesh] OR "Evidence Based Practice"[tw] OR "Evidence-Based Health Care"[tw] OR "Evidence Based Health Care"[tw] OR "Evidence-Based Health Cares"[tw] OR "Health Care, Evidence-Based"[tw] OR "Health Cares, Evidence-Based"[tw] OR "Evidence-Based Healthcare"[tw] OR "Evidence Based Healthcare"[tw] OR "Evidence-Based Healthcares"[tw] OR "Healthcare, Evidence-Based"[tw] OR "Healthcares, Evidence-Based"[tw] OR "Evidence Based Management, Healthcare"[tw] OR "Evidence Based Health Care Management"[tw] OR "Evidence Based Healthcare Management"[tw] OR "Evidence Based Management, Health Care"[tw])) OR ("Quality Indicators, Health Care"[Mesh] OR "Quality Indicators, Healthcare"[tw] OR "Healthcare Quality Indicator"[tw] OR "Healthcare Quality Indicators"[tw] OR "Indicator, Healthcare Quality"[tw] OR "Indicators, Healthcare Quality"[tw] OR "Quality Indicator, Healthcare"[tw] OR "Health Metrics"[tw] OR "Health Metric"[tw] OR "Metrics, Health"[tw] OR "Global Trigger Tool, Healthcare"[tw] OR "Healthcare Global Trigger Tool"[tw])) OR ("Quality of Health Care"[Mesh] OR "Health Care Quality"[tw] OR "Quality of Healthcare"[tw] OR "Healthcare Quality"[tw] OR "Quality of Care"[tw] OR "Care Quality"[tw] OR "Pharmacy Audit"[tw] OR "Audit, Pharmacy"[tw] OR "Pharmacy Audits"[tw])) OR ("Patient Generated Health Data"[Mesh] OR "Self-Recorded Health Data"[tw] OR "Data, Self-Recorded Health"[tw] OR "Health Data, Self-Recorded"[tw] OR "Health Datas, Self-Recorded"[tw] OR "Self Recorded Health Data"[tw] OR "Patient Generated Clinical Data"[tw] OR "Patient Generated Data"[tw] OR "Data, Patient Generated"[tw] OR "Generated Data, Patient"[tw])) OR ("Disease Eradication"[Mesh] OR "Disease Eradications"[tw] OR "Eradication, Disease"[tw] OR "Eradications, Disease"[tw] OR "Disease Elimination"[tw] OR "Disease Eliminations"[tw] OR "Elimination, Disease"[tw] OR "Eliminations, Disease"[tw])) OR ("Health Education"[Mesh] OR "Education, Health"[tw] OR "Community Health Education"[tw] OR "Education, Community Health"[tw] OR "Health Education, Community"[tw])) OR ("Technology Transfer"[Mesh] OR "Technology Transfers"[tw] OR "Transfer, Technology"[tw] OR "Transfers, Technology"[tw] OR "Technology Licensing"[tw] OR "Licensing, Technology"[tw] OR "Licensings, Technology"[tw] OR "Technology Licensings"[tw] OR "Research Commercialization"[tw] OR "Commercialization, Research"[tw] OR "Commercializations, Research"[tw] OR "Research Commercializations"[tw] OR "Technology Commercialization"[tw] OR "Commercialization, Technology"[tw] OR "Commercializations, Technology"[tw] OR "Technology Commercializations"[tw])) OR ("Public Health Informatics"[Mesh] OR "Informatics, Public Health"[tw])) OR ("Health Literacy"[Mesh] OR "Literacy, Health"[tw])) OR ("Quality Assurance, Health Care"[Mesh] OR "Healthcare Quality Assurance"[tw] OR "Assurance, Healthcare Quality"[tw] OR "Assurances, Healthcare Quality"[tw] OR "Healthcare Quality Assurances"[tw] OR "Quality Assurances, Healthcare"[tw] OR "Quality Assurance, Healthcare"[tw] OR "Healthcare Quality Assessment"[tw] OR "Assessment, Healthcare Quality"[tw] OR "Assessments, Healthcare Quality"[tw] OR "Healthcare Quality Assessments"[tw] OR "Quality Assessments, Healthcare"[tw] OR "Quality Assessment, Healthcare"[tw] OR "Quality Assessment, Health Care"[tw] OR "Health Care Quality Assessment"[tw])) OR ("Patient Acceptance of Health Care"[Mesh] OR "Health Care Utilization"[tw] OR "Utilization, Health Care"[tw] OR "Patient Acceptance of Healthcare"[tw] OR "Healthcare Patient Acceptance"[tw] OR "Healthcare Patient Acceptances"[tw] OR "Nonacceptors of Health Care"[tw] OR "Care Nonacceptor, Health"[tw] OR "Care Nonacceptors, Health"[tw] OR "Health Care Nonacceptor"[tw] OR "Health Care Nonacceptors"[tw] OR "Health Care Seeking Behavior"[tw] OR "Acceptors of Health Care"[tw] OR "Care Acceptor, Health"[tw] OR "Care Acceptors, Health"[tw] OR "Health Care Acceptor"[tw] OR "Health Care Acceptors"[tw] OR "Health Care Acceptability"[tw] OR "Acceptability of Healthcare"[tw] OR "Healthcare Acceptabilities"[tw] OR "Healthcare Acceptability"[tw])) OR ("Needs Assessment"[Mesh] OR "Needs Assessments"[tw] OR "Educational "[tw] OR "Assessment, Educational Needs"[tw] OR ", Educational"[tw] OR "Needs Assessments, Educational"[tw] OR "Determination of Health Care Needs"[tw] OR "Determination of Healthcare Needs"[tw] OR "Assessment of Healthcare Needs"[tw] OR ", Healthcare"[tw] OR "Needs Assessments, Healthcare"[tw])) OR ("Health Care Evaluation Mechanisms"[Mesh] OR "Healthcare Evaluation Mechanisms"[tw] OR "Evaluation Mechanism, Healthcare"[tw] OR "Evaluation Mechanisms, Healthcare"[tw] OR "Healthcare Evaluation Mechanism"[tw] OR "Mechanism, Healthcare Evaluation"[tw] OR "Mechanisms, Healthcare Evaluation"[tw])) OR ("Delivery of Health Care"[Mesh] OR "Delivery of Healthcare"[tw] OR "Healthcare Deliveries"[tw] OR "Healthcare Delivery"[tw] OR "Deliveries, Healthcare"[tw] OR "Delivery, Healthcare"[tw] OR "Health Care Delivery"[tw] OR "Delivery, Health Care"[tw] OR "Contraceptive Distribution"[tw] OR "Contraceptive Distributions"[tw] OR "Distribution, Contraceptive"[tw] OR "Distributions, Contraceptive"[tw] OR "Delivery of Dental Care"[tw] OR "Dental Care Delivery"[tw] OR "Delivery, Dental Care"[tw] OR "Health Care"[tw] OR "Care, Health"[tw] OR "Healthcare"[tw] OR "Health Care Systems"[tw] OR "Health Care System"[tw] OR "System, Health Care"[tw] OR "Systems, Health Care"[tw] OR "Healthcare Systems"[tw] OR "Healthcare System"[tw] OR "System, Healthcare"[tw] OR "Systems, Healthcare"[tw] OR "Nonclinical Distribution"[tw] OR "Distributions, Nonclinical"[tw] OR "Nonclinical Distributions"[tw] OR "Distribution, Nonclinical"[tw] OR "Distribution, Non-Clinical"[tw] OR "Distribution, Non Clinical"[tw] OR "Distributions, Non-Clinical"[tw] OR "Non-Clinical Distributions"[tw] OR "Non-Clinical Distribution"[tw] OR "Non Clinical Distribution"[tw] OR "Community-Based Distribution"[tw] OR "Community Based Distribution"[tw] OR "Community-Based Distributions"[tw] OR "Distribution, Community-Based"[tw] OR "Distributions, Community-Based"[tw] OR "Distributional Activities"[tw] OR "Activities, Distributional"[tw] OR "Activity, Distributional"[tw] OR "Distributional Activity"[tw])) OR ("Disease Management"[Mesh] OR "Disease Managements"[tw] OR "Management, Disease"[tw] OR "Managements, Disease"[tw])) OR ("Health Care Surveys"[Mesh] OR "Care Survey, Health"[tw] OR "Care Surveys, Health"[tw] OR "Health Care Survey"[tw] OR "Survey, Health Care"[tw] OR "Surveys, Health Care"[tw] OR "Healthcare Surveys"[tw] OR "Healthcare Survey"[tw] OR "Survey, Healthcare"[tw] OR "Surveys, Healthcare"[tw] OR "National Hospital Discharge Survey"[tw] OR "National Ambulatory Medical Care Survey"[tw])) OR ("Delivery of Health Care, Integrated"[Mesh] OR "Integrated Health Care Systems"[tw] OR "Integrated Delivery Systems"[tw] OR "Delivery System, Integrated"[tw] OR "Delivery Systems, Integrated"[tw] OR "Integrated Delivery System"[tw] OR "System, Integrated Delivery"[tw] OR "Systems, Integrated Delivery"[tw])) OR ("Health Care Quality, Access, and Evaluation"[Mesh] OR "Healthcare Quality, Access, and Evaluation"[tw])) OR ("Process Assessment, Health Care"[Mesh] OR "Assessment, Process"[tw] OR "Assessments, Process"[tw] OR "Process Assessment"[tw] OR "Process Assessments"[tw] OR "Process Assessment (Health Care)"[tw] OR "Assessment, Process (Health Care)"[tw] OR "Assessments, Process (Health Care)"[tw] OR "Process Assessments (Health Care)"[tw] OR "Process Measures"[tw] OR "Measure, Process"[tw] OR "Measures, Process"[tw] OR "Process Measure"[tw])) OR ("Outcome Assessment, Health Care"[Mesh] OR "Outcome Assessment"[tw] OR "Outcomes Assessment"[tw] OR "Outcome Assessment (Health Care)"[tw] OR "Assessment, Outcome (Health Care)"[tw] OR "Assessments, Outcome (Health Care)"[tw] OR "Outcome Assessments (Health Care)"[tw] OR "Assessment, Outcomes"[tw] OR "Assessments, Outcomes"[tw] OR "Outcomes Assessments"[tw] OR "Assessment, Outcome"[tw] OR "Assessments, Outcome"[tw] OR "Outcome Assessments"[tw] OR "Outcomes Research"[tw] OR "Research, Outcomes"[tw] OR "Outcome Studies"[tw] OR "Outcome Study"[tw] OR "Studies, Outcome"[tw] OR "Study, Outcome"[tw] OR "Outcome Measures"[tw] OR "Measure, Outcome"[tw] OR "Measures, Outcome"[tw] OR "Outcome Measure"[tw])) OR ("Health Systems Agencies"[Mesh] OR "Agencies, Health Systems"[tw] OR "Agency, Health Systems"[tw] OR "Health Systems Agency"[tw] OR "Systems Agencies, Health"[tw] OR "Systems Agency, Health"[tw] OR "Comprehensive Health Planning Agencies"[tw])))) AND (((((((((("Pakistan"[Mesh] OR Islamic Republic of Pakistan[tw]) OR ("Uganda"[Mesh] OR Republic of Uganda[tw])) OR ("Sudan"[Mesh] OR Republic of the Sudan[tw])) OR ("Bangladesh"[Mesh] OR bangladesh[tw])) OR ("Ethiopia"[Mesh] OR Federal Democratic Republic of Ethiopia[tw])) OR ("Lebanon"[Mesh] OR "Lebanese Republic"[tw])) OR ("Democratic Republic of the Congo"[Mesh] OR "Congo (Kinshasa)"[tw] OR "Zaire"[tw] OR "Belgian Congo"[tw] OR "Katanga"[tw])) OR ("Kenya"[Mesh] OR "Republic of Kenya"[tw])) OR ("Cameroon"[Mesh] OR "Republic of Cameron"[tw] OR "United Republic of Cameroon"[tw] OR "Cameroons"[tw])) OR ("Iran"[Mesh] OR "Islamic Republic of Iran"[tw]))

All of the above terms were combined using the PubMed search filter for randomized controlled trials and participatory studies. The compiled search terms were then adapted for use with Embase and WoS in combination with their database-specific filters for paper type. The searches were re-run before initial and final paper analysis for inclusion to ensure the most current research was presented in our review.

# **Bias Assessments**

## *Publication Bias*

Journals that published more than one article were studied for publication bias. The JAMA and the European Journal: Tropical Medicine & International Health had 50% positive and 50% negative results. Transactions of the Royal Society of Tropical Medicine and Hygiene had the next best distribution of publication outcomes, with 50% of their studies having positive outcomes, 33% negative, and 17% mixed, followed by PLoS and the Lancet with 60% positive outcomes and 63% positive outcomes, respectively. Two-thirds of the published studies in the Malaria Journal and BMJ had positive outcomes, and both of BMC's published studies had positive outcomes.

## *Study Risk of Bias*

As detailed in the main text, we assessed the studies’ risk of bias using the Mixed Methods Appraisal Tool (MMAT).[^3^](https://www.zotero.org/google-docs/?n3RYah) MMAT as quality assessments for five study designs: qualitative research, randomized controlled trials, non-randomized studies, quantitative descriptive studies, and mixed methods studies. Following initial screening, articles are categorized based on article type and screened with their respective questions. MMAT discourages reviewers from creating an overall score. The format of the tool is depicted in the figure below.


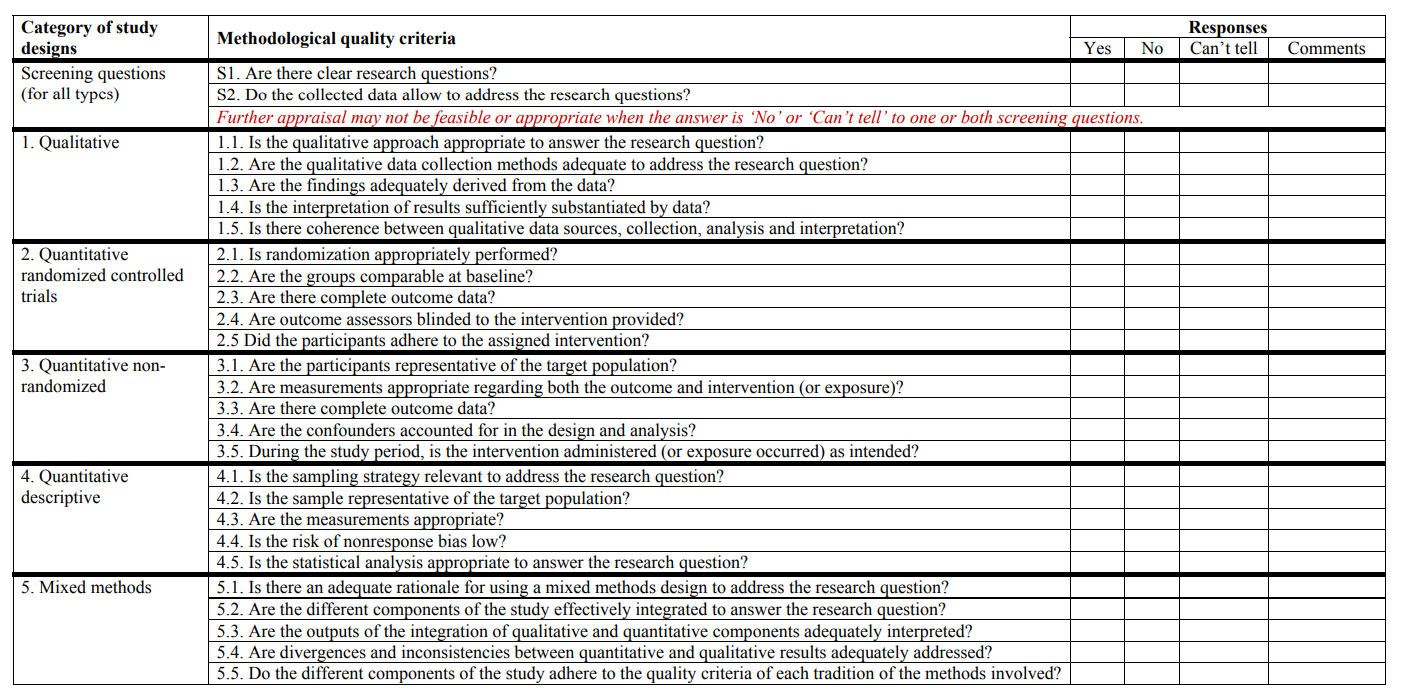


**Figure S1.** MMAT Risk of Bias Questionnaire.

This review included two mixed methods studies. Both studies provided adequate rationale for using this study design while integrating different components of qualitative and quantitative research into their hypothesis. The outputs of each component were sufficiently integrated into the results, and both addressed any divergences between the components. Only one study in this sample did not adhere to the quality criteria of the methods they had originally set forth. Figure S2 depicts the reviewers' scoring.


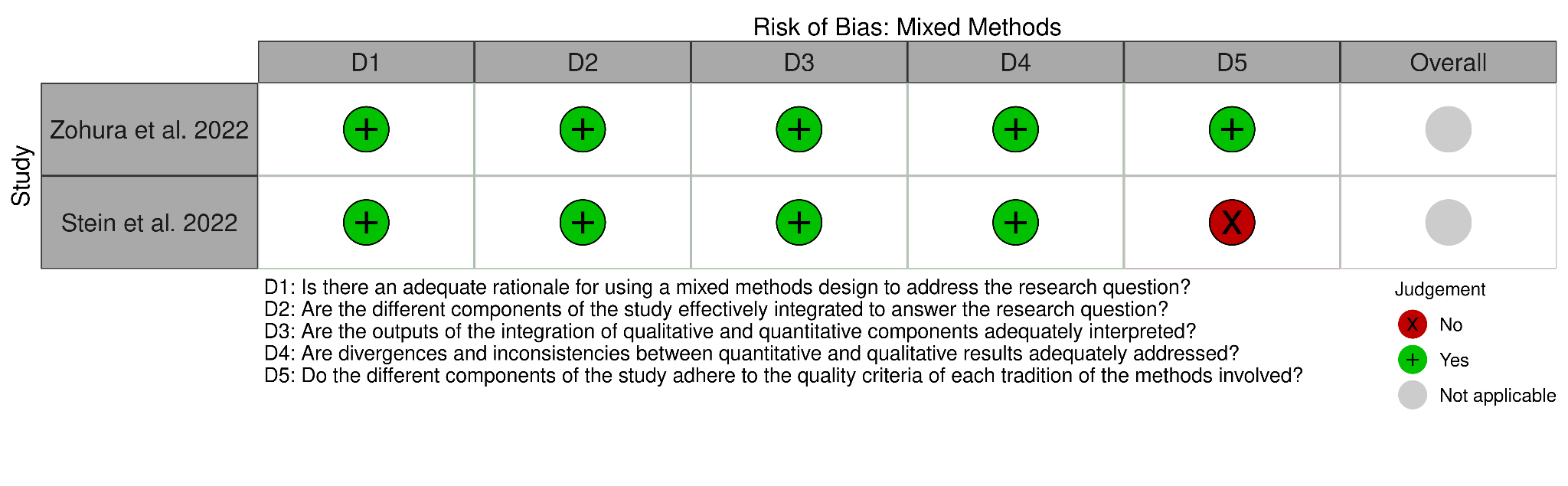


**Figure S2**. MMAT Mixed Methods results.

This review had only one qualitative study, which met all of the criteria imposed by MMAT. Briefly, they adequately described why the qualitative approach was used and subsequently used an appropriate data collection method. Furthermore, the findings and interpretations were both substantiated by the data, and there was a consistency between the sourcing, collection, analysis, and interpretation of the data. Figure S3 depicts the final decision between the MMAT reviewers.


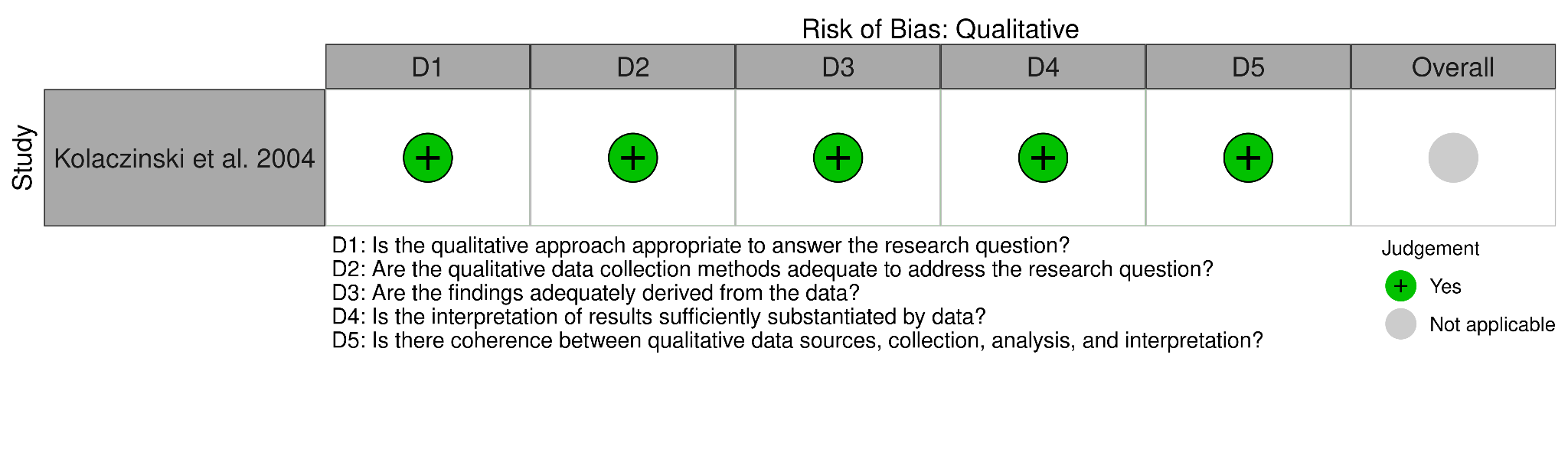


**Figure S3**. MMAT Qualitative results.

Of the forty included studies, six were quantitative descriptive studies. One study had a risk of bias from a high potential of nonresponse bias. The other studies all provided sufficient information that was relevant to the research question, representative of the target population, and appropriately measured variables. Every study detailed and justified its statistical measurements. Figure S4 depicts the scoring of quantitative descriptive analyses.


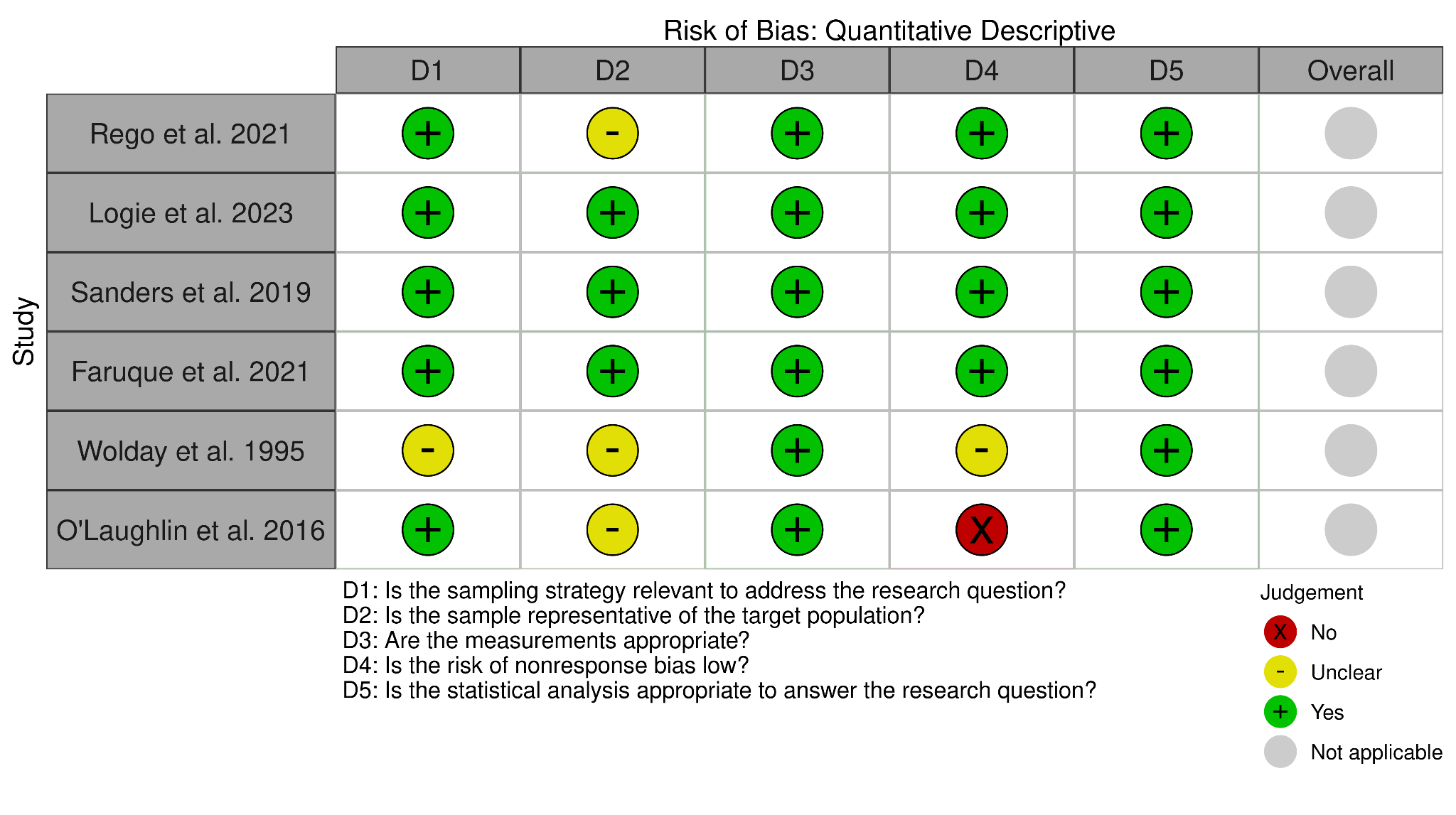


**Figure S4**. MMAT Quantitative Descriptive results.

Two quantitative non-randomized control studies were included in this study. Both studies included participants who were representative of the population and appropriately measured the outcome and intervention. In one study, it was unclear whether researchers had appropriately accounted for potential confounders. Both interventions were administered as intended. Figure S5 depicts the scoring given by the reviewers.


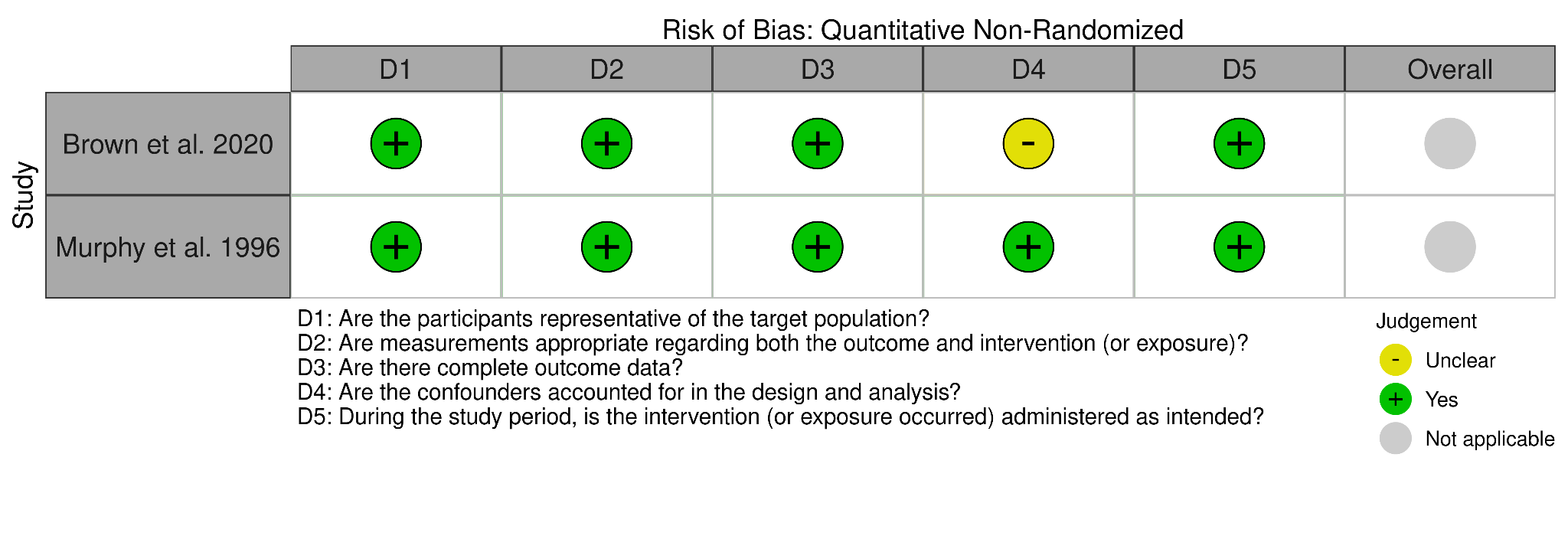


**Figure S5.** MMAT Quantitative Non-Randomized results.

Over half of the articles included in this review (29, 72.5%) were Quantitative Randomized Controlled Trials. Two studies did not adequately conduct their randomization process adequately. In several of the studies, it was unclear whether the intervention and control groups were comparable at baseline. Only one study did not have complete outcome data due to 67% of the initial study population dying or moving out of the study area. Nine studies were not blinded, and eight studies had unclear blinding of the outcome assessor(s). Finally, four studies had people “not adhere” to the intervention. In one study, several participants washed clothes that were treated with an insecticide to prevent malaria transmission after instructions not to do so. With many of the studies that tested bed nets, we were not able to determine whether participants adequately adhered to the intervention. Figure S6 depicts the final type of study within the risk of bias assessment.


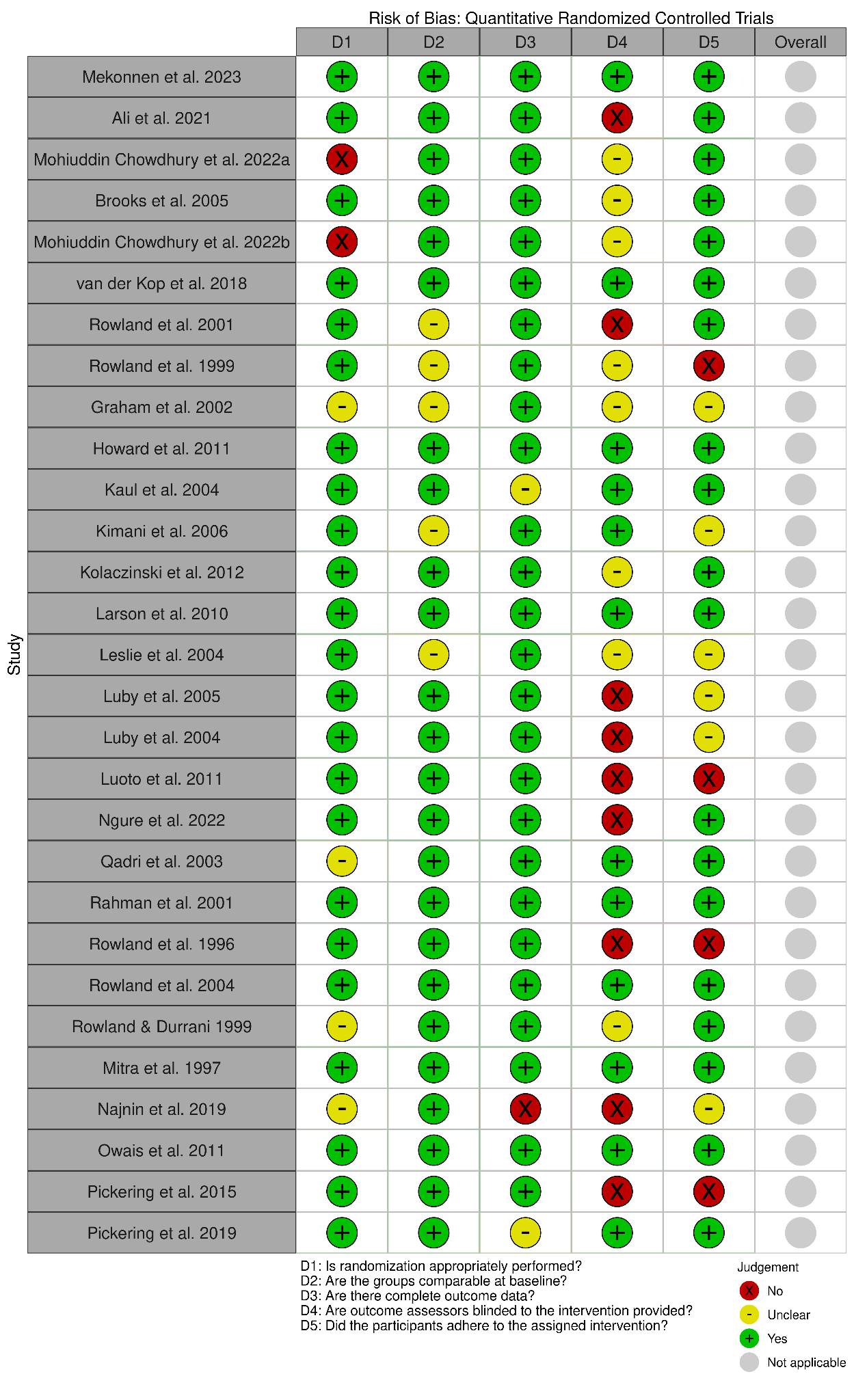


**Figure S6.** MMAT Quantitative Randomized Control Trial results.

# **Post Abstract Screening Cuts**

Studies that were included after the first round of screening but were excluded after the full-text screening are included in Table S1 with exclusion rationale.

**Table S1**. Post abstract screening cuts.

| **Paper** | **Reason** |
| --- | --- |
| Synder et al., 2020[^4^](https://www.zotero.org/google-docs/?Xgo5BA): The sustainability and scalability of private sector sanitation delivery in urban informal settlement schools: A mixed methods follow-up of a randomized trial in Nairobi, Kenya | Not infectious disease-related |
| Haque et al., 2010[^5^](https://www.zotero.org/google-docs/?2ttF1u): Low-dose β-carotene supplementation and deworming improve serum vitamin A and β-carotene concentrations in preschool children of Bangladesh | Not infectious disease-related |
| De Vita et al., 2019[^6^](https://www.zotero.org/google-docs/?RPHKDE): Malnutrition, morbidity, and infection in the informal settlements of Nairobi, Kenya: an epidemiological study | Not a primary analysis |
| Kang et al., 2022[^7^](https://www.zotero.org/google-docs/?CbE7JM): Are better existing WASH practices in urban slums associated with a lower long-term risk of severe cholera? A prospective cohort study with 4 years of follow-up in Mirpur, Bangladesh | Not a primary analysis |
| Bella et al., 2019[^8^](https://www.zotero.org/google-docs/?BMwiZy): Trachoma prevalence in refugee camps in the East Region of Cameroon | Full text unavailable |
| Soares et al., 1996[^9^](https://www.zotero.org/google-docs/?XtC9Pz): Efficacy of bacillare dysentery's treatment by lomefloxacine amongst Rwandese refugees in North Zaire | No English translation (originally in French) |
| Turan et al., 2012[^10^](https://www.zotero.org/google-docs/?SpjbRE): The study of HIV and antenatal care integration in pregnancy in Kenya: Design, methods, and baseline results of a cluster-randomized controlled trial | No mention of target population |
| Soares et al., 1994[^11^](https://www.zotero.org/google-docs/?QcbwQL): Short-term ciprofloxacin treatment of bacillary dysentery due to Shigella dysenteriae type 1 in Rwandan refugees | Full text unavailable |
| Salih and Mohamed, 2021[^12^](https://www.zotero.org/google-docs/?BnEDrq): A case analysis of a mass treatment approach to control GI and water-related conditions in Sudan | Not a primary analysis |
| Rotheram-Borus et al., 2012[^13^](https://www.zotero.org/google-docs/?5MfkbD): Vocational Training with HIV Prevention for Ugandan Youth | Not infectious disease-related |
| Bermudez et al., 2019[^14^](https://www.zotero.org/google-docs/?s2Sdpf): HIV risk among displaced adolescent girls in Ethiopia: The role of gender attitudes and self-esteem | Not a primary analysis |
| Logie et al., 2022[^15^](https://www.zotero.org/google-docs/?Dal63M): Todurujo na Kadurok (empowering youth): study protocol of an HIV self-testing and edutainment comic cluster randomized trial among refugee youth in a humanitarian setting in Uganda | Study protocol |
| Mumma et al., 2019[^16^](https://www.zotero.org/google-docs/?HJQdJ6): The Safe Start trial to assess the effect of an infant hygiene intervention on enteric infections and diarrhea in low-income informal neighborhoods of Kisumu, Kenya: a study protocol for a cluster randomized controlled trial | Study protocol |
| Solmeimani-Ahmadi et al., 2012[^17^](https://www.zotero.org/google-docs/?9vVMSS): Field evaluation of permethrin long-lasting insecticide treated nets (Olyset®) for malaria control in an endemic area, southeast of Iran | No mention of target population |
| Solmeimani-Ahmadi et al., 2012[^18^](https://www.zotero.org/google-docs/?y27w7S): Effects of educational intervention on long-lasting insecticidal nets use in a malarious area, southeast Iran | No mention of target population |
| Smith et al., 2021[^19^](https://www.zotero.org/google-docs/?sadpPk): Effective demand for in-line chlorination bundled with rental housing in Dhaka, Bangladesh | Not infectious disease-related |
| Sarkar et al., 2002[^20^](https://www.zotero.org/google-docs/?UwESpx): Effect of deworming on nutritional status of Ascaris infected slum children of Dhaka, Bangladesh | Not infectious disease-related |
| Okumu et al., 2022[^21^](https://www.zotero.org/google-docs/?ChAUwn): Support for texting-based condom negotiations among forcibly displaced adolescents in the slums of Kampala, Uganda: Cross-sectional validation of the Condom Use Negotiated Experiences Through Technology Scale | Not infectious disease-related |
| Ali et al., 2019[^22^](https://www.zotero.org/google-docs/?a1E8Ww): Unmasking herd protection by an oral cholera vaccine ina cluster-randomized trial | Not a primary analysis |
| Halder et al., 2023[^23^](https://www.zotero.org/google-docs/?r3im7d): COVID-19 preventative measures in Rohingya refugee camps: An assessment of knowledge, attitude and practice | Preprint |

#

#

#

#

#

#

#

#

#

#

#

# **References**

[1 Larkin KH. MeSH Miner. https://hahahammond.pythonanywhere.com/meshminer](https://www.zotero.org/google-docs/?a7pYRq)

[2 UNHCR. Refugee host countries by income level. UNHCR Refug. Stat. 2023. https://www.unhcr.org/refugee-statistics/insights/explainers/refugee-host-countries-income-level.html (accessed Jan 9, 2024).](https://www.zotero.org/google-docs/?a7pYRq)

[3 Hong Q, Pluye P, Fàbregues, S, *et al.* Mixed Methods Appraisal Tool (MMAT), version 2018. 2018.](https://www.zotero.org/google-docs/?a7pYRq)

[4 Snyder JS, Prentice-Mott G, Boera C, Mwaki A, Alexander KT, Freeman MC. The Sustainability and Scalability of Private Sector Sanitation Delivery in Urban Informal Settlement Schools: A Mixed Methods Follow Up of a Randomized Trial in Nairobi, Kenya. *Int J Environ Res Public Health* 2020; **17**: 5298.](https://www.zotero.org/google-docs/?a7pYRq)

[5 Haque R, Ahmed T, Wahed MA, Mondal D, Rahman ASMH, Albert MJ. Low-dose β-carotene Supplementation and Deworming Improve Serum Vitamin A and β-carotene Concentrations in Preschool Children of Bangladesh. *J Health Popul Nutr* 2010; **28**: 230–7.](https://www.zotero.org/google-docs/?a7pYRq)

[6 De Vita MV, Scolfaro C, Santini B, *et al.* Malnutrition, morbidity and infection in the informal settlements of Nairobi, Kenya: an epidemiological study. *Ital J Pediatr* 2019; **45**: 12.](https://www.zotero.org/google-docs/?a7pYRq)

[7 Kang S, Chowdhury F, Park J, *et al.* Are better existing WASH practices in urban slums associated with a lower long-term risk of severe cholera? A prospective cohort study with 4 years of follow-up in Mirpur, Bangladesh. *BMJ Open* 2022; **12**: e060858.](https://www.zotero.org/google-docs/?a7pYRq)

[8 Bella A, Coulibaly S, Nko’Ayissi G, *et al.* Trachoma prevalence in refugee camps in the East Region of Cameroon. *Am J Trop Med Hyg* 2019; **101**: 1–668.](https://www.zotero.org/google-docs/?a7pYRq)

[9 Soares JL, Milleliri JM, Pigny N, Dupoux J, Coue JC. Efficacy of bacillare dysentery’s treatment by lomefloxacine amongst Rwandese refugees in North Zaire. *Médecine Mal Infect* 1996; **26**: 141–4.](https://www.zotero.org/google-docs/?a7pYRq)

[10 Turan JM, Steinfeld RL, Onono M, *et al.* The Study of HIV and Antenatal Care Integration in Pregnancy in Kenya: Design, Methods, and Baseline Results of a Cluster-Randomized Controlled Trial. *PLOS ONE* 2012; **7**: e44181.](https://www.zotero.org/google-docs/?a7pYRq)

[11 Soares JL, Arendt V, Coue JC, *et al.* Short-term ciprofloxacin treatment of bacillary dysentery due to Shigella dysenteriae type 1 in Rwandan refugees. *Med Trop Rev Corps Sante Colon* 1994; **54**: 319–23.](https://www.zotero.org/google-docs/?a7pYRq)

[12 Salih A, Mohamed M. A case analysis of a mass treatment approach to control GI and water-related conditions in Sudan. *BMC Public Health* 2021; **21**: 2111.](https://www.zotero.org/google-docs/?a7pYRq)

[13 Rotheram-Borus MJ, Lightfoot M, Kasirye R, Desmond K. Vocational Training with HIV Prevention for Ugandan Youth. *AIDS Behav* 2012; **16**: 1133–7.](https://www.zotero.org/google-docs/?a7pYRq)

[14 Bermudez LG, Yu G, Lu L, *et al.* HIV Risk Among Displaced Adolescent Girls in Ethiopia: the Role of Gender Attitudes and Self-Esteem. *Prev Sci Off J Soc Prev Res* 2019; **20**: 137–46.](https://www.zotero.org/google-docs/?a7pYRq)

[15 Logie CH, Okumu M, Loutet MG, *et al.* Todurujo na Kadurok (empowering youth): study protocol of an HIV self-testing and edutainment comic cluster randomised trial among refugee youth in a humanitarian setting in Uganda. *BMJ Open* 2022; **12**: e065452.](https://www.zotero.org/google-docs/?a7pYRq)

[16 Mumma J, Simiyu S, Aseyo E, *et al.* The Safe Start trial to assess the effect of an infant hygiene intervention on enteric infections and diarrhoea in low-income informal neighbourhoods of Kisumu, Kenya: a study protocol for a cluster randomized controlled trial. *BMC Infect Dis* 2019; **19**: 1066.](https://www.zotero.org/google-docs/?a7pYRq)

[17 Soleimani-Ahmadi M, Vatandoost H, Shaeghi M, *et al.* Field evaluation of permethrin long-lasting insecticide treated nets (Olyset(®) for malaria control in an endemic area, southeast of Iran. *Acta Trop* 2012; **123**: 146–53.](https://www.zotero.org/google-docs/?a7pYRq)

[18 Soleimani-Ahmadi M, Vatandoost H, Shaeghi M, *et al.* Effects of educational intervention on long-lasting insecticidal nets use in a malarious area, southeast Iran. *Acta Med Iran* 2012; **50**: 279–87.](https://www.zotero.org/google-docs/?a7pYRq)

[19 Smith DW, Sultana S, Crider YS, *et al.* Effective Demand for In-Line Chlorination Bundled with Rental Housing in Dhaka, Bangladesh. *Environ Sci Technol* 2021; **55**: 12471–82.](https://www.zotero.org/google-docs/?a7pYRq)

[20 Sarkar NR, Anwar KS, Biswas KB, Mannan MA. Effect of deworming on nutritional status of ascaris infested slum children of Dhaka, Bangladesh. *Indian Pediatr* 2002; **39**: 1021–6.](https://www.zotero.org/google-docs/?a7pYRq)

[21 Okumu M, Logie CH, Ansong D, Mwima S, Hakiza R, Newman PA. Support for Texting-Based Condom Negotiation Among Forcibly Displaced Adolescents in the Slums of Kampala, Uganda: Cross-sectional Validation of the Condom Use Negotiated Experiences Through Technology Scale. *JMIR Public Health Surveill* 2022; **8**: e27792.](https://www.zotero.org/google-docs/?a7pYRq)

[22 Ali M, Qadri F, Kim DR, *et al.* Unmasking herd protection by an oral cholera vaccine in a cluster-randomized trial. *Int J Epidemiol* 2019; **48**: 1252–61.](https://www.zotero.org/google-docs/?a7pYRq)

[23 Halder CE, Hasan MA, Mohamed Y, *et al.* COVID-19 preventive measures in Rohingya refugee camps: An assessment of knowledge, attitude and practice toward COVID-19. 2023; : 2023.02.21.23286227.](https://www.zotero.org/google-docs/?a7pYRq)
